# Slow Noise in the Period of a Biological Oscillator Underlies Gradual Trends and Abrupt Transitions in Phasic Relationships in Hybrid Neural Networks

**DOI:** 10.1371/journal.pcbi.1003622

**Published:** 2014-05-15

**Authors:** Umeshkanta S. Thounaojam, Jianxia Cui, Sharon E. Norman, Robert J. Butera, Carmen C. Canavier

**Affiliations:** 1 Department of Cell Biology and Anatomy, Louisiana State University Health Sciences Center New Orleans, Louisiana, United States of America; 2 BioCircuits Institute, University of California, San Diego, La Jolla, California, United States of America; 3 School of Electrical and Computer Engineering, Georgia Institute of Technology, Atlanta, Georgia, United States of America; 4 Department of Biomedical Engineering, Georgia Institute of Technology and Emory University, Atlanta Georgia, United States of America; 5 Neuroscience Center of Excellence, Louisiana State University Health Sciences Center, New Orleans, Louisiana, United States of America; École Normale Supérieure, France

## Abstract

In order to study the ability of coupled neural oscillators to synchronize in the presence of intrinsic as opposed to synaptic noise, we constructed hybrid circuits consisting of one biological and one computational model neuron with reciprocal synaptic inhibition using the dynamic clamp. Uncoupled, both neurons fired periodic trains of action potentials. Most coupled circuits exhibited qualitative changes between one-to-one phase-locking with fairly constant phasic relationships and phase slipping with a constant progression in the phasic relationships across cycles. The phase resetting curve (PRC) and intrinsic periods were measured for both neurons, and used to construct a map of the firing intervals for both the coupled and externally forced (PRC measurement) conditions. For the coupled network, a stable fixed point of the map predicted phase locking, and its absence produced phase slipping. Repetitive application of the map was used to calibrate different noise models to simultaneously fit the noise level in the measurement of the PRC and the dynamics of the hybrid circuit experiments. Only a noise model that added history-dependent variability to the intrinsic period could fit both data sets with the same parameter values, as well as capture bifurcations in the fixed points of the map that cause switching between slipping and locking. We conclude that the biological neurons in our study have slowly-fluctuating stochastic dynamics that confer history dependence on the period. Theoretical results to date on the behavior of ensembles of noisy biological oscillators may require re-evaluation to account for transitions induced by slow noise dynamics.

## Author Summary

Many biological phenomena exhibit synchronized oscillations in the presence of noise and heterogeneity. These include brain rhythms that underlie cognition and spinal rhythms that underlie rhythmic motor activity like breathing and locomotion. A two oscillator system was constructed in which most of the circuit was implemented in a computer model, and was therefore completely known and under the control of the investigators. The one biological component was an oscillator in which an apparently novel manifestation of biological noise was identified, dynamical noise in the period of the oscillator itself. This study quantifies how much noise and heterogeneity this simple two oscillator system can tolerate before desynchronizing. More complicated systems of oscillators may follow similar principles.

## Introduction

Synchronized neural firing is a characteristic activity pattern of neural systems. Synchronized neural activity in cortical circuits [1] is thought to underlie many aspects of cognition [2], [3], including recognition [4], recall [5], perception [6], [7], and attention [8]. Phase-locked neural activity is also an essential component of central pattern generators (CPGs) located in the spinal cords of vertebrates and the ganglia of invertebrates [9], [10]. Inhibition plays a central role in oscillatory synchrony, and in this study we focus on reciprocal inhibitory coupling.

A major contribution of this paper is a distinct notion of noise in coupled oscillatory neurons, which we explore by comparing three models of noise intrinsic to the neurons (see Methods). The dominant source of noise in neurons is thought to be synaptic [11]. This thinking is shaped by studies of cortical circuits, in which neurons in a high conductance state that receive a stochastic barrage of fast and balanced excitatory and inhibitory input show fast fluctuations in membrane potential [12]. An early attempt to quantify the effect of noise on neural activity [13] examined the case of a perfect integrator with additive white noise. The output of the integrator is interpreted as the membrane potential. In the absence of noise, a baseline current produces a regular oscillator with constant angular velocity that is reset each time it reaches threshold. The noise takes the form of Gaussian current noise added to the baseline current. When this noise is integrated, it is analogous to a trajectory produced by Brownian motion, and produces a one-dimensional random walk in the membrane potential superimposed on the steady upward trend caused by the constant baseline current. In this model, membrane potential is proportional to the phase of the oscillation, so a random walk in the phase occurs. The time scale of this noise is fast, due to its theoretically flat spectrum which includes very high frequency components. The current noise has no history-dependence since the value at each time point is random and independent of all previous values. However, the membrane potential does have a memory, because at each time step, the value is a perturbed version of the value at the previous time step. The second moment, or variance, of the displacement of the membrane potential from its original value is proportional to the product of the diffusion constant and the time step. The mean squared displacement therefore grows as the square root of the size of the time step [14]. The memory of noise on the previous cycle is wiped out when the membrane potential and the phase are reset when the spike threshold is reached. Based on this model, a common way to add noise to phase models of neurons is simply to add Gaussian noise to the phase [15], [16], which is one of the noise models that we use in this study.

Real neurons have complex nonlinear intrinsic currents, and thus may not linearly integrate their extrinsic inputs. We modeled the intrinsic period as stochastic due to random fluctuations in factors that influence the period. If these factors have little history dependence, for example, variability in the number of ions passing through an open channel at any given time, then successive interspike intervals are uncorrelated and may appear to be drawn from a Gaussian distribution [13], [17], [18]. Gaussian noise added to the period is the second model used in this study. If the period of one cycle depends on the previous cycle because the stochastic fluctuations occur in history-dependent processes, then a different model must be used [19]. History dependent noise may arise from slowly changing levels of stochastic fluctuations in the numbers of open channels for adaptation currents [20] or levels of second messengers, channel phosphorylation, insertion and deletion of channels into the membrane, and other unknown factors. Instead of drawing the period from a distribution, the period itself can be made to undergo Brownian motion under the assumption that the period is equally likely to be perturbed in either direction at a given instant, and that the displacement is therefore proportional to the square root of the time step. Finally, if we assume that the mean of the noise reverts to zero, we obtain an Ornstein-Uhlenbeck [21] process added to a constant period, which is the third and final noise model used in this study. This latter model is novel, although it shares some elements with the model of Schwalger et al. [20], and constitutes a different noise model that may complement the fast noise in some circumstances. We postulate that the period of biological oscillatory neurons varies randomly but with history dependence. The direct effect on network activity of slow stochastic dynamics that cause history dependence in the period of component oscillators has not been previously investigated. This slow form of intrinsic noise may have implications for synchronization and phase locking in neural circuits.

In this study, we construct hybrid neural circuits consisting of one biological and one computationally modeled neuron. These coupled pairs exhibit different patterns of activity, which we refer to as motifs, during coupling. Understanding how and why synchronization and phase locking occur in populations of neurons is critical to understanding how neural circuits function. Phase-locking implies a constant phase relationship between neural oscillators; synchrony is a special case of phase-locking in which spikes occur in different neurons at about the same time. Another observed motif is phase slipping. In this motif, the spiking activity of the faster cell “laps” the slower one and the timing relationships are different in every cycle. Our analysis of these dynamics utilizes the phase resetting curve (PRC) measured from both biological and model neurons in response to the same stimulus pulses that the neurons receive in the circuit; an action potential in the presynaptic neuron triggers a predetermined conductance waveform in the postsynaptic neuron both in the hybrid network and in the protocol for measuring the PRC. The PRC describes how a neuron's period is shortened or lengthened depending upon at what point in the cycle a perturbation was received [22], [23]. This PRC is a useful tool for predicting synchronization and phase locking in neural systems under the assumption that the phase resetting due to an input is complete by the time the neuron receiving the input spikes next or by the time it receives another input, whichever occurs first. The PRC for biological neurons as well as the hybrid circuit activity is measured in the presence of ubiquitous biological noise. The impact of noise on PRC-based predictions is an open question.

The overall aim of this work was to assess why different dynamical motifs, such as phase locking and phase slipping, were observed in hybrid circuits and to explain how random transitions between these motifs occurred. Using PRC-based maps, we were able to predict phase locking and synchronization in two-neuron networks and describe the activity motifs observed in these circuits. By comparing the performance of three noise models in simulations of hybrid circuit activity, we were able to show that noise contributes to variability within and switching between different motifs, and that history-dependent noise in the period was necessary to mimic motif variability and transitions seen in experiments.

## Materials and Methods

### *Aplysia californica* preparation

*Aplysia californica* were acquired from the Miami National Resource for Aplysia (Miami, FL) and kept in saltwater tanks at room temperature for 1–2 weeks until used. Animals were anesthetized using a solution of 71.2 g MgCl_2_ in 1 L 1X artificial sea water (1X ASW). 1X ASW was comprised of (in mM) 460 NaCl, 10 KCl, 11 CaCl_2_, 30 MgCl_2_, 25 MgSO_4_, and 10 HEPES (pH 7.6) [24]. The abdominal ganglion was dissected out of the animal and pinned in a Sylgard-lined (Dow Corning) dish filled with dissection solution (30% 1X MgCl_2_ solution and 70% ASW solution) for desheathing. The ganglion was desheathed under a dissection microscope. The dish solution was then replaced with a high-Mg^2+^ low Ca ^2+^ recording solution, which contained (in mM) 330 NaCl, 10 KCl, 90 MgCl_2_, 20 MgSO_4_, 2 CaCl_2_, and 10 HEPES, pH 7.6 [25]. Electrodes consisted of pulled (Sutter P-97 puller) glass pipettes containing 3 M potassium acetate and silver wire chlorided in bleach.

Regularly spiking neurons in the lower left quadrant of the *Aplysia* were used as the biological neurons in the hybrid circuits. An Axoclamp 2B amplifier with Clampex 8.2 software (Molecular Devices) was used to supply stimulus currents and record membrane potential. A Digidata 1322A Digitizer (Molecular Devices) was used to sample electrophysiological data at 10 kHz.

### Model neurons

Wang-Buzsaki (WB) model neurons were used in the hybrid circuit experiments. The equations and parameters for the WB model neuron were the same as in [26] except that the leak reversal potential E_L_ was set to -60.0 mV and the applied current I_app_ was controlled to match the 1–5 Hz spiking frequency of the *Aplysia* spiking neuron. This modified WB model matches both the spike dynamics and PRC shape of experimentally measured neurons [27], [28]. I_app_ for the model neuron was chosen such that the spiking frequency was similar to that of the biological neuron. Synaptic conductance values for the model neuron were selected to increase the likelihood of 1∶1 phase locking in hybrid circuits. The differential equations for the state variables of the WB model and the two virtual synapses were updated in real time. The voltage measured in the biological neuron was used to determine the time course of the conductance for the synapse onto the model neuron and the driving force for the synaptic current of the synapse onto the biological neuron.

### Dynamic clamp

Dynamic clamp is a real-time computational and experimental technique used to add data-driven simulated ion channel conductances to biological neurons [29]–[31]. For these experiments, we used the Model Reference Current Injection (MRCI) [32] system to construct hybrid circuits and measure phase resetting curves. The dynamic clamp system operated at a frequency of 10 kHz, which corresponds to a closed-loop sampling and computation period of 100 µs. Reciprocal inhibitory synapses were used in hybrid circuits, and inhibitory perturbations were used to measure phase resetting curves. The alpha-shaped conductance waveform was calculated using the following equations: *dy/dt  =  −y/τ +itrig*; dα/dt  =  −α*/τ + y; I_syn_  = g_syn_* α*(V- E_syn_) e*/*τ*. *V* corresponds to the membrane potential of the postsynaptic cell, *E_syn_* was set to −70 mV, and *τ* and *g_syn_* were varied as in Table 1. The value of *itrig* was zero except when an input was triggered, either because the presynaptic cell spiked in the hybrid circuit or a perturbation was needed to measure a point on the PRC, then *itrig* was set to amplitude 1 for 1 ms. The *e*/*τ* term normalizes the maximum amplitude of the conductance waveform to *g_syn_*.

**Table 1:** Summary of experimental data.

Exp.#	Bio.	R^2^	g_MB_	g_BM_	Peak_B_	Peak_M_	τ	P_B_	P_M_	ISI_B_	ISI_M_
1	1	0.012	0.03	0.45	0.0659	0.1063	10	558.98	545.7	568.15	574.96
2	2	0.063	0.03	0.28	0.0452	0.0406	10	285.16	285.8	287.34	290.22
3	3	0.136	0.05	0.5	0.0399	0.0396	5	353.62	349.1	368.73	359.11
4	4	0.276	0.07	0.9	0.0442	0.1497	5	729.9	799.3	788.07	837.53
5	4	0.339	0.04	0.3	0.0431	0.0925	10	767.9	728.3	801.90	780.78
6	5	0.373	0.04	1.92	0.0879	0.7759	25	482.5	478.6	485.61	573.36
7	6	0.381	0.05	0.5	0.0371	0.0786	5	680.84	672.9	673.05	684.9
8	4	0.400	0.04	0.3	0.0683	0.2004	20	783.68	799.3	803.73	883.24
9	7	0.408	0.03	10.0	0.0461	0.8292	10	248.34	235.6	289.56	289.44
10	4	0.462	0.07	0.9	0.0754	0.29	10	805.44	799.3	846.42	850.32
11	8	0.485	0.04	0.15	0.1021	0.1464	15	1593.6	1457.1	1595.83	1611.8
12	9	0.499	0.03	0.45	0.0386	0.0852	10	380.58	383.9	387.21	391.69
13	10	0.519	0.05	1.0	0.1190	0.2516	15	379.5	374.3	378.57	393.46
14	9	0.527	0.04	0.6	0.0627	0.0482	5	324.9	316.0	331.87	328.38
15	10	0.585	0.05	1.0	0.0482	0.0902	5	409.56	405.4	409.34	410.95
16	3	0.589	0.05	0.5	0.0465	0.0631	7.5	378.97	374.3	389.48	389.71
17	2	0.618	0.03	0.9	0.0452	0.1244	10	273.89	266.0	292.256	290.07
18	2	0.693	0.03	4.0	0.0452	0.5113	10	272.56	266.0	279.15	284.56
19	4	0.814	0.04	0.3	0.0431	0.1007	10	838.82	799.3	868.05	865.47
20	2	0.845	0.03	5.0	0.0452	0.6048	10	275.2	266.0	280.11	282.13
21	5	0.854	0.04	1.92	0.0161	0.1766	5	425.7	417.5	428.96	431.12
22	5	0.87	0.04	1.92	0.0555	0.4923	15	439.7	417.5	430.83	442.83
23	8	0.933	0.04	0.15	0.0650	0.1796	20	1262.9	1417.5	1641.6	1666.6
24	10	0.939	0.06	1.0	0.0579	0.0947	5	391.0	383.9	397.47	397.93
25	4	0.945	0.04	0.3	0.0528	0.1507	15	830.8	799.3	871.65	870.56
26	10	0.95	0.03	5.0	0.0289	0.4379	5	444.78	445.1	457.36	458.01
27	2	0.954	0.03	0.8	0.0452	0.0828	10	272.46	266.0	280.24	272.17
28	4	0.954	0.07	1.2	0.0754	0.3592	10	875.19	856.7	855.26	867.42
29	11	0.954	0.02	2.0	0.0542	0.3002	10	351.8	334.7	362.39	362.74
30	5	0.957	0.04	1.92	0.0675	0.6256	20	424.14	417.5	486.77	480.5
31	12	0.957	0.02	2.0	0.0179	0.1997	10	190.52	176.5	194.86	194.5
32	5	0.958	0.04	1.92	0.0366	0.3092	10	396.3	365.3	421.40	423.77
33	13	0.961	0.04	0.4	0.0789	0.131	10	795.45	779.7	823.06	828.73
34	5	0.964	0.07	1.2	0.0435	0.1891	5	806.3	761.4	832.02	838.12
35	10	0.975	0.05	0.8	0.1189	0.1959	15	369.64	361.1	382.17	381.58

Experiments are numbered in increasing order of R^2^ value. “Bio” indicates the biological neuron corresponding to each experiment. The synaptic conductance from model neuron to biological neuron g_MB_ is given in mS, whereas the synaptic conductance from biological to model neuron g_BM_ is given in µS/cm^2^. Peak_B_ and Peak_M_ give the maximum phase resetting for biological and model neurons respectively. Since the model and experimental neurons have different conductance conventions, the peaks are a better indicator of relative coupling strength. The synaptic time constant τ was the same for both neurons and is given in ms. P_B_ and P_M_ are the uncoupled periods in ms for the biological and model neurons, respectively. ISI_B_ and ISI_M_ are the average network ISIs in ms for the biological and model neurons, respectively, calculated from the experimental data during the course of coupling episode.

### Phase resetting curves

**Figure 1:**
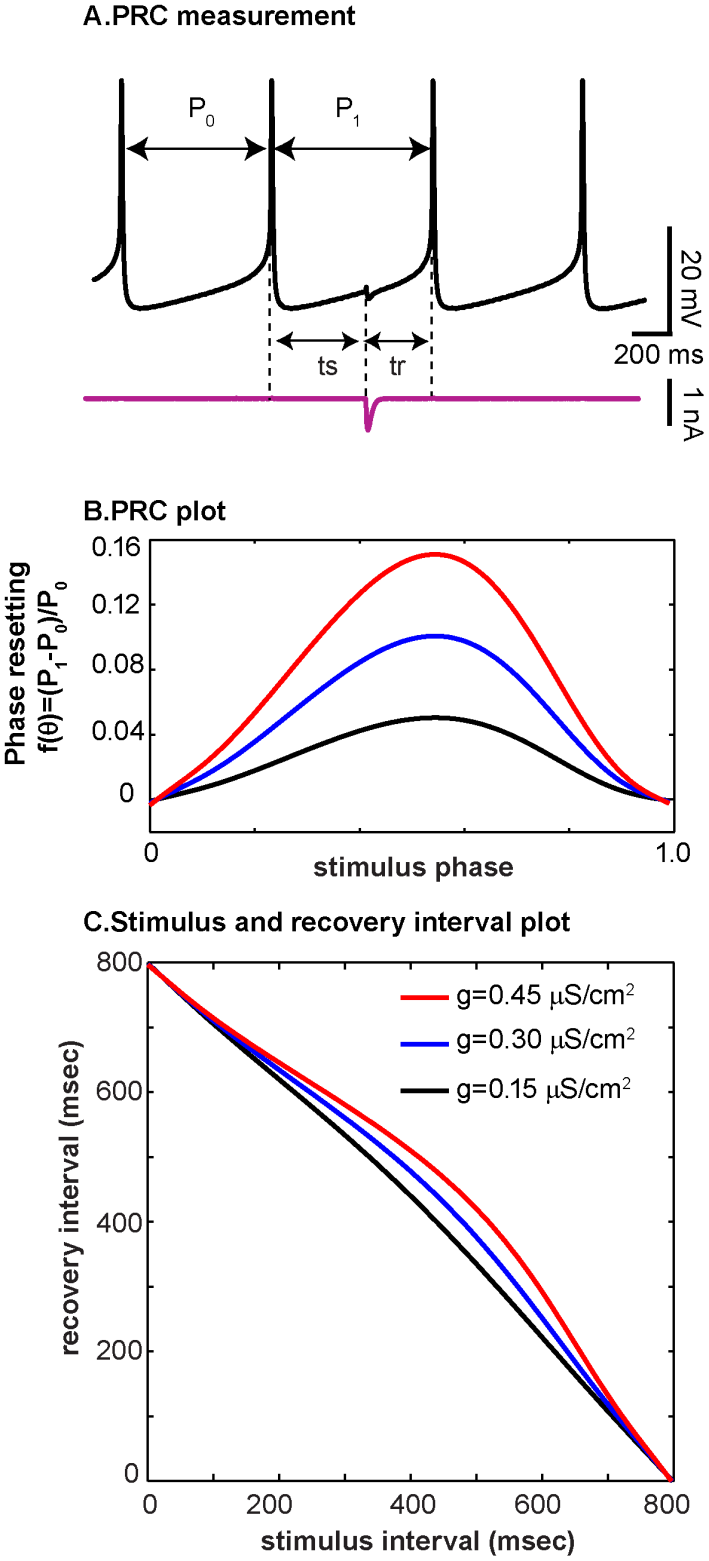
Phase resetting curve measurement and interval mapping. (A) Illustration of how inhibitory perturbations are applied at various intervals after the reference spike, using a trace from the biological neuron as measured in experiment 19. (B) Phase resetting curves are plotted as stimulus phase *ts/P_0_* vs phase reset *(P_1_-P_0_)/P_0_* for different inhibitory synaptic conductance strengths (µS/cm^2^) given in the inset to panel C. The blue trace is a PRC measured in the model neuron from experiment 19, using the dynamic clamp operating at 10 kHz with g_syn_ = 0.3 µS/cm^2^. The estimated PRCs for g_syn_ = 0.15 and 0.45 µS/cm^2^ were approximated by scaling the measured PRC. (C) Information from the PRC can be plotted instead as recovery interval (*tr*) vs. stimulus interval (*ts*). Notice that PRCs with substantial curvature can appear relatively flat in the *ts-tr* plane.

PRCs were measured using the dynamic clamp to apply inhibitory inputs at various times during the neuron's interspike interval (ISI). Perturbations were separated by at least 10 cycles to allow ISIs to return to pre-perturbation magnitudes. The stimulus interval *ts* corresponds to the time interval between the previous spike in the neuron receiving the input and the start of the applied perturbation. This interval was normalized by unperturbed period P_0_, which was the average of the five ISIs prior to the perturbation, to obtain the phase *θ = ts/P_0_*. Phase reset (Figure 1B) was calculated as the perturbed period *P_1_* minus the unperturbed period P_0_, normalized by the unperturbed period *P_0_* (see Figure 1A). Neuronal spikes were detected using a −40 mV threshold. Biological PRCs were fit using 3^rd^ or 4^th^ order polynomials to minimize least squared error and promote randomly-distributed residuals. Noiseless model neuron PRCs were spline fit. This fit was necessary in order to use the PRCs as functions in the network simulations described below.

An alternative way to present the information from a PRC is in the stimulus interval – recovery interval (*ts-tr*) plane (Figure 1C). Stimulus interval refers to the time interval between when the neuron last spiked and when a perturbation arrived. The recovery interval *tr* refers to the interval between the time of application of the perturbation and the time of the next spike in the perturbed neuron. This description preserves time information, unlike the PRC whose quantities are unitless. Very strong perturbations result in more pronounced curves on the *ts-tr* plane, whereas less strong perturbations manifest in the *ts-tr* plane as nearly straight lines. As seen in Figure 1B and C, a PRC with peak magnitude of around 0.05 (black curve), looks somewhat like a straight line on the *ts-tr* plane. This apparent flattening occurs because the PRC plot is scaled to the maximum PRC amplitude, whereas the scale of the *ts-tr* plot is determined by the maximum period of the oscillation.

### Hybrid circuits

Hybrid circuits of one biological neuron and one model neuron were constructed using the dynamic clamp; 13 distinct biological neurons were used to construct the 35 hybrid circuits presented here. No noise was added to the circuit, all noise was intrinsic to the biological neuron. A single biological neuron was used for multiple hybrid circuits, with different conductance and time constant values, for as long as the experiment remained viable. All synapses were inhibitory because the reversal potential for both synapses (*E_syn_*) was set to −70 mV. In nearly all cases, PRCs of the biological neurons were measured with conductance parameters g_syn_ and τ that were used for the coupling experiments. In a limited number of cases, coupling experiments were performed with a weaker conductance than the one at which the corresponding PRC was measured. In such cases, the PRC was linearly scaled to calculate the curves that describe the network interactions. We previously showed that for conductance below a certain threshold, PRC shape is preserved and scales linearly with amplitude [33]. Our goal was to choose coupling values that resulted in 1∶1 synchrony; however, because the PRC measured before the experiment constrains the coupling parameters used in the experiment, but the biological neuron activity can change over time, in practice a range of effective couplings were obtained.

**Figure 2:**
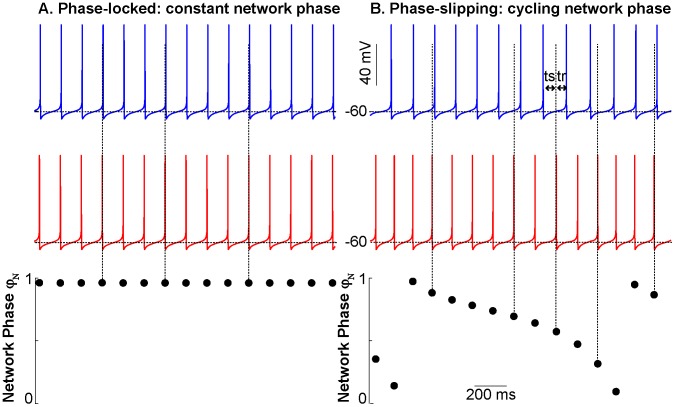
Schematic illustration of phase-locking and slipping motifs in two-neuron networks. The top traces show the membrane potential traces of two coupled model neurons, whereas the bottom trace shows the network phase for every spike in the red neuron relative to the spikes in the blue neuron that bracket it. Vertical dashed lines show the point within the cycle of the blue neuron at which a spike in the red neuron occurs. (A) One neuron spikes consistently at the same point within the interspike interval of the other neuron. Phase-locking occurs and coupled neurons have a consistent, stable phase relationship. (B) One neuron spikes faster than the other neuron and in this case, phase slipping occurs. The spike in the red trace occurs at progressively earlier points during the interspike interval between the blue spikes until a blue interspike interval actually contains two red spikes.

Dynamical motifs were defined as characteristically different episodes of network activity. Network phase φ_net_ was defined as the position of the spike in the biological neuron within the cycle in the model neuron that contains the spike. Network phase was calculated as *ts_M_/(ts_M_ + tr_M_)*, where *ts_M_* is the time interval between a spike in the model neuron and the following spike in the biological neuron (which perturbs the model neuron), and *tr_M_* is the time interval between the spike in the biological neuron and the next spike in the model neuron. The first 10 network phases were discarded to eliminate transient effects. Network phase that remained within ±0.1 units of the network phase for 20 or more cycles was defined as phase-locked (Figure 2A). Activity in which the network phase transitioned through consecutive increasing or decreasing phases, which often resulted in one neuron spiking twice during the ISI of the other neuron, was defined as phase slipping (Figure 2B). Episodes that did not meet either criterion were categorized as other. See Text S1 for more detailed information on the algorithm used for automated characterization. In some cases, coupling was turned on and off during an experiment; this was done to determine the robustness of the hybrid circuit activity.

To measure the consistency of phase locking, we used circular statistics to find the R^2^ metric, often referred to as the vector strength, for each experiment [34]. In circular statistics, values are represented by a unit vector and an angle. The average vector captures the mean angle φ_ave_ of all the data and the magnitude R, which is a measure of the tightness of the locking [35]. In our case, the average network phase is φ_ave_ for the phase of the firing of the model neuron within the cycle of the biological neuron, and the magnitude R corresponds to how consistent the network phase is during an experiment. The strength of phase locking is represented by the length of the vector, *R*, where *R^2^ = X^2^+Y^2^*. As in [34], φ_ave_ and R^2^ are calculated using
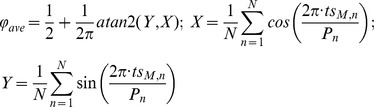
where atan2 is the two argument arctan function that returns a value between –π and π, P_n_ is the network period measured in cycle n, N is the number of network periods, and *ts_M,n_* is the n^th^ stimulus time for neuron M, the model cell. Note that the signs of X and Y must be considered in the two argument version of arctan to put φ_ave_ in the appropriate quadrant. In [34], an R^2^ threshold of 0.7 is used to distinguish strongly phase-locked systems, which have R^2^ near 1, from those with weaker locking. Higher R^2^ magnitudes indicate that a system does not deviate much from the phase-locked angle and has a dominant phase-locked mode, while lower R^2^ magnitudes indicate more variability in network phase. R^2^ calculations and PRC fits were performed in MATLAB (The MathWorks).

### Network simulations and noise models

Each hybrid circuit experiment was simulated using PRC-based maps. In these simulations, the phase variable evolves at a rate determined by the intrinsic frequency, with instantaneous phase resetting applied at the time of input from the other neuron according to the measured PRCs. A key assumption is that the shape of the PRC does not change with the relatively small changes in the period of the oscillator. Simulated PRCs were constructed to mimic the shape and magnitude of biological and model neurons used during experiments. Network simulations were performed in C. Conceptually, our noiseless map [36], [37] is a modified Winfree [22] phase model in which the intrinsic phase *θ_i_* ranges from 0 to 1, and is reset from 1 to 0 when a spike occurs 
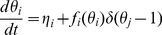
(1)where *η_i_* is the angular velocity in neuron *i*, *θ_j_* is the phase in presynaptic neuron *j* and *f_i_(θ_i_)* is the phase resetting due to each spike in presynaptic neuron *j.* We do not integrate Equation 1, instead we assume the phase changes at a constant velocity between inputs, and jumps instantaneously when an input is received. The result is a coupled nonlinear map, which was used to simulate both the PRC experiments and the hybrid circuit experiments and implemented as follows. The map requires the PRC and the initial value of the intrinsic period for each neuron, and the initial values of the phase of each neuron. The phases are only updated at the times associated with each episode of neural firing, so the first step after initialization is to determine which neuron(s) will fire next. This is accomplished by finding the shortest recovery interval (*tr_i_ = P_i_(1- θ_i_))*, where P_i_ is the current estimate of intrinsic period of the i^th^ neuron, based on the noise models given in the main text, and *θ_i_* is its phase. At the next firing time, the phase of the firing neuron is reset to zero. The recovery interval in the next neuron (*j*) to fire is also the stimulus interval (*ts_i_*) for the nonfiring neuron (*i*). The phase of neuron *i* is calculated as *θ_i_ = ts_i_/P_i_* and the phase is decremented by the resetting *f_i_(θ_i_)* calculated at that phase when a spike occurs in the presynaptic neuron *j*. The next event is again determined by finding the shortest recovery interval (*tr_j_ = P_j_(1- θ_j_)*) until the next spike. We added noise to Eq. 1 model in three ways, which renders it a Langevin equation in phase.

#### i) Gaussian noise added directly to the PRC

We added Gaussian noise to the fitted PRC of the biological neuron by updating its phase by *f_B_(θ_B_) +σX* where *X* is a normally distributed random variable N(0,1) and σ is the standard deviation. When noise is added to the PRC, the maximum effective standard deviation of the intrinsic period change is given by *σ_eff_  =  μ σ/(1+f_B,min_(θ_B_))*, where μ is the average period measured prior to turning on the coupling and *f_B,min_(θ_B_)* is the minimum phase resetting for the biological neuron PRC (see Text S1 for details). Therefore the phase resetting in Eq. 1 has both a deterministic component and a stochastic component with no history dependence in this scheme.

#### ii) Gaussian noise added directly to the mean intrinsic period

We added Gaussian noise to the intrinsic period of the biological neuron on each cycle so that *tr_B_ = (P_B_+ σP_B_X)(1- θ_B_)* and *θ_B_ = ts_B_/(P_B_+σ P_B_X)*. The effective standard deviation of the period itself is given by σ_eff_  = μ σ. Since η_i_ in Eq. 1 is the inverse of the period (*P_B_+σ P_B_X*), η_B_ is a random process with no history dependence in this scheme.

#### iii) Ornstein-Uhlenbeck process in the period

Instead of considering the intrinsic period as a constant, the period of the biological neuron was allowed to vary as an Ornstein-Uhlenbeck process [21] in which the fast random perturbations to the period can accumulate over a slow time scale that pulls the period back to the mean. This causes η_B_ in Eq. 1 to be a random process with history dependence in this scheme. The history dependence is the critical distinction.
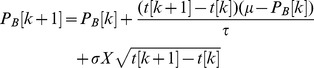
(2)where the index *k* denotes each successive sampling time *t[k]*, *P_B_[k]* is the estimate of the intrinsic period at that time, *X* is a Gaussian random variable N(0,1), μ is the average period measured prior to turning on the coupling, σ is the standard deviation, and τ is the time constant for mean reversion. The phase is calculated as *θ_B_ = ts_B_/P_B_[k]* and the recovery interval as *tr_B_ = P_B_[k](1- θ_B_*). In this case, the effective standard deviation of the period over a long period of time is given by 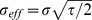 [38]. An alternate way to interpret the above equation is to define a slow noise variable *y[k]  = P_B_[k]* – μ that is added to the mean period to produce the history dependent variability in the period.

A new estimate of the instantaneous limit cycle velocity (*1/P_B_[k+1]*) is sampled whenever a neuron spikes or an input is received, and is assumed to be constant over the interval between samples *t[k+1]-t[k]*. There are three possible cases in which this occurs:

- We know *t[k+1]-t[k]* because it is equal to the stimulus interval *ts*, which is the interval between the last spike and the arrival of an input. This case can occur either in the simulations of PRC measurement or the simulation of the hybrid circuit.
- We do not know *t[k+1]-t[k]* because it is equal to the recovery interval *tr*, which is the interval between the arrival of an input and the next spike, and is also the quantity we are trying to predict. The deterministic value of *tr* was used as an approximation, and calculated directly from *ts* using the previous estimate (*P_B_[n]*) before the pull towards the mean or the random diffusion component was applied.
- We do not know *t[k+1]-t[k]* because it is equal to *P_B_[k+1]*, which is the very quantity we want to estimate. This situation only occurs when there is no input during a cycle, as when we simulate the five unperturbed cycles that are interspersed between each PRC measurement. The previous value *P_B_[k]* is used as an estimate for *t[k+1]-t[k]*.

The critical difference between the first two noise models and the third one is that the first two simply involve jittering the resetting or the period, whereas in the third model, the jitter accumulates from cycle to cycle and is pulled back to the mean on a slow time scale. The last two noise models require the assumption that the shape of the PRC does not change significantly as the period is varied over the range covered by the experiment. All noise models required the computation of the mean period μ, which was set to the average of 5 unperturbed cycles preceding each experiment (PRC or coupled circuit) in order to match the experimental protocol. For the OU simulations, *P_B_[k]* was also initialized with this value.

In order to obtain σ for the case of Gaussian noise added to the PRC or mean period, the PRC was simulated by adding noise to the map. Five unperturbed cycles were allowed to elapse between each perturbation, and the average of those noisy observed periods was used to calculate the phase from the stimulus interval at about twenty evenly spaced intervals, as was done for the experiments. The parameter σ was adjusted until the squared error (SE) of the noisy simulated PRC with respect to the smooth polynomial fit was approximately equal to that of the experimental data. The Ornstein-Uhlenbeck model required an extra parameter and an iterative procedure. After σ was set using the PRC protocol, hybrid circuit simulations were performed for 10 random seeds for the same duration of coupling as observed in each experiment. The initial value of *τ* was set to 1000 times the value of the period of biological neuron given in Table 1. A bifurcation was defined as a transition between phase locking and phase slipping, and the value of *τ* was adjusted until the range of number of bifurcations observed in the simulations bracketed the value observed experimentally. Then the procedure was repeated until both criteria were satisfied. Parameters for the three noise models for each experiment, the range of bifurcations, and the SE ratio for simulations to experiments are given in Table 2.

**Table 2:** Comparison of noise models.

Experiment		OU				Noise added to PRC			Noise added to period		
#	NB	σ,τ (sec)	μ/τ	SE Ratio	NB	σ	SE Ratio	NB	σ	SE Ratio	NB
1	2	(0.435, 588.98)	0.001	0.9939	[0,3]	0.0284	0.9971	[0,0]*	0 .0193	0.9962	[0,0]*
2	3	(0.0671, 285.16)	0.001	0.9910	[0,3]	0.00378	0.9940	[0,0]*	0 .00641	0.994	[0,0]*
3	4	(0.118, 3.5362)	0.1	0.9934	[0,6]	0.00875	0.9992	[0,0]*	0.00879	0.9941	[9], [11]*
4	1	(0.0935, 79.990)	0.01	0.99813	[0,3]	0.00349	0.9958	[0,0]*	0.00545	0.9961	[0,0]*
5	1	(0.0414, 767.9)	0.001	0.9935	[0,3]	0.00127	0.9935	[1], [4]	0.00212	0.9939	[0,0]*
6	3	(0.2155, 48.25)	0.01	0.99809	[0,3]	0.0097	0.9988	[0,3]	0 .01595	0.9946	[0,0]*
7	1	(0.1457, 680.84)	0.001	0.9939	[0,3]	0.00499	0.9916	[0,0]*	0 .00856	0.9976	[0,0]*
8	4	(0.1195,78.368)	0.01	0.9937	[0,6]	0.00695	0.9925	[0,0]*	0.00678	0.9921	[0,4]
9	12	(0.1455,2.4834)	0.01	0.9888	[6], [12]	0.00579	0.9940	[0,0]*	0 .0064	0.9997	[0,0]*
10	2	(0.0738,80.544)	0.01	0.9951	[0,3]	0 .00251	0.9979	[0,0]*	0 .00335	0.9916	[0,0]*
11	1	(0.511, 159.357)	0.01	0.9928	[0,2]	0.01291	0.9928	[0,0]*	0.0194	0.9918	[0,0]*
12	2	(0.0499, 38.058)	0.01	0.9913	[0,5]	0.00244	0.9939	[0,0]*	0.00405	0.9966	[0,0]*
13	1	(0.101, 37.952)	0.01	0.99684	[0,4]	0.00792	0.9948	[0,0]*	0.00737	0.9958	[0,0]*
14	4	(0.0996,3.249)	0.1	0.9895	[3], [6]	0.00481	0.9968	[0,0]*	0.00747	0.9946	[0,5]*
15	3	(0.0743, 40.956)	0.01	0.9969	[1], [5]	0 .00605	0.9929	[0,0]*	0 .00568	0.9921	[0,0]*
16	0	(0.1594,3.78)	0.1	0.9775	[0,6]	0.0112	0.9981	[0,0]	0.0118	0.9941	[0,0]
17	5	(0.0666, 27.389)	0.01	0.9995	[0,9]	0.00378	0.9940	[0,0]*	0.00636	0.9954	[0,0]*
18	5	(0.0591, 27.256)	0.01	0.9744	[2], [8]	0.00383	0.9948	[0,0]*	0.00609	0.9967	[0,0]*
19	2	(0.0453, 83.882)	0.01	0.9958	[0,3]	0.00142	0.9937	[0,0]*	0.00213	0.9979	[0,0]*
20	4	(0.0688,27.520)	0.01	0.9924	[3], [12]	0.0039	0.9905	[0,0]*	0.00637	0.9910	[0,0]*
21	1	(0.232,4.257)	0.1	0.9902	[0,5]	0.0088	0.9918	[0,0]*	0. 01491	0.9940	[0,0]*
22	5	(0.211, 43.97)	0.01	0.9910	[0,6]	0.00953	0.9939	[0,0]*	0.0149	0.9901	[0,0]*
23	1	(0.885,12.629)	0.1	0.9957	[0,2]	0.0231	0.9933	[0,0]*	0.0390	0.9947	[0,0]*
24	0	(0.0965, 3.91)	0.1	0.9966	[0,1]	0.00741	0.9961	[0,0]	0.00698	0.9909	[0,0]
25	0	(0.1986, 8.308)	0.1	0.9936	[0,1]	0.0109	0.9975	[0,0]	0.01	0.9812	[0,0]
26	0	(0.513,4.4478)	0.1	0.9952	[0,4]	0.00364	0.9952	[0,0]	0.00379	0.9947	[0,0]
27	0	(0.075, 2.7246)	0.1	0.9832	[0,1]	0.00418	0.9942	[0,0]	0.00634	0.9905	[0,0]
28	0	(0.1982,8.7519)	0.1	0.9984	[0,2]	0 .00799	0.9925	[0,0]	0 .0118	0.9963	[0,0]
29	0	(0.355, 0.3158)	0.1	0.99068	[0,4]	0 .0191	0.9938	[0,0]	0 .01769	0.9903	[0,0]
30	0	(0.121,42.414)	0.01	0.9954	[0,4]	0 .01315	0.9938	[0,0]	0 .0203	0.9987	[0,0]
31	0	(0.1075, 1.9052)	0.1	0.9908	[0,0]	0 .00626	0.9923	[0,0]	0 .011085	0.9965	[0,0]
32	0	(0.1687, 3.963)	0.1	0.9955	[0,0]	0 .00677	0.9966	[0,0]	0 .0117	0.9937	[0,0]
33	0	(0.2685, 7.9545)	0.01	0.9969	[0,0]	0 .00924	0.9974	[0,0]	0 .0149	0.9920	[0,0]
34	0	(0.1047,80.63)	0.01	0.9996	[0,0]	0.00379	0.9942	[0,0]	0.00532	0.9987	[0,0]
35	0	(0.0987, 3.6964)	0. 1	0. 9967	[0,0]	0.00767	0.9984	[0,0]	0.0075	0.9906	[0,0]

For each experiment number (#), the number of bifurcations (NB) is given under the heading Experiment. For each noise model, including Ornstein Uhlenbeck (OU) and Gaussian noise added to the PRC or period, the model parameters are given under the appropriate headings. For the OU model, the ratio of the mean period to the OU time constant is also given. For each noise model, the squared error (SE) between the simulated PRC and the best fit to the experimental data was calculated, and the ratio of this error to that observed in the experimentally measured PRC was adjusted to be close to one without exceeding one. This ratio is given in the table for each model. Finally, the range of bifurcations observed in simulations of network phase using 10 random seeds is given in brackets. An asterisk beside the bracket indicates model failure, because the simulations failed to bracket the experimentally observed value within the constraints imposed by the noise calibration.

## Results

### Coupled neurons show well-defined dynamical motifs in phase relationships

To construct hybrid circuits, one biological neuron from the abdominal ganglion of *Aplysia californica* was reciprocally coupled to one Wang-Buzsaki (WB) [26] conductance-based model neuron using the dynamic clamp [29], [30]. The dynamic clamp measures the potential in the biological neuron, integrates the differential equations for the WB model and the two virtual synapses, and injects synaptic current into the biological neuron. The WB model was used because it produces phase resetting curves (PRCs) that are comprised of only delays in response to an inhibitory input (Figure 1B), and because the WB PRCs resemble those measured in *Aplysia* neurons [27], [28]. Parameters for the hybrid circuits and maximum phase resetting values for the biological and model neurons are shown in Table 1. Notice that the maximum phase resetting is different between the biological and model neurons; this discrepancy creates a heterogeneous system. The average interspike interval of the biological neuron during coupling, which corresponds to the network period if the system is phase-locked, is different than the uncoupled biological neuron period; this provides evidence that the motifs observed in our hybrid networks result from mutual coupling effects, and do not reflect entrainment of the model neuron by the biological neuron.

**Figure 3:**
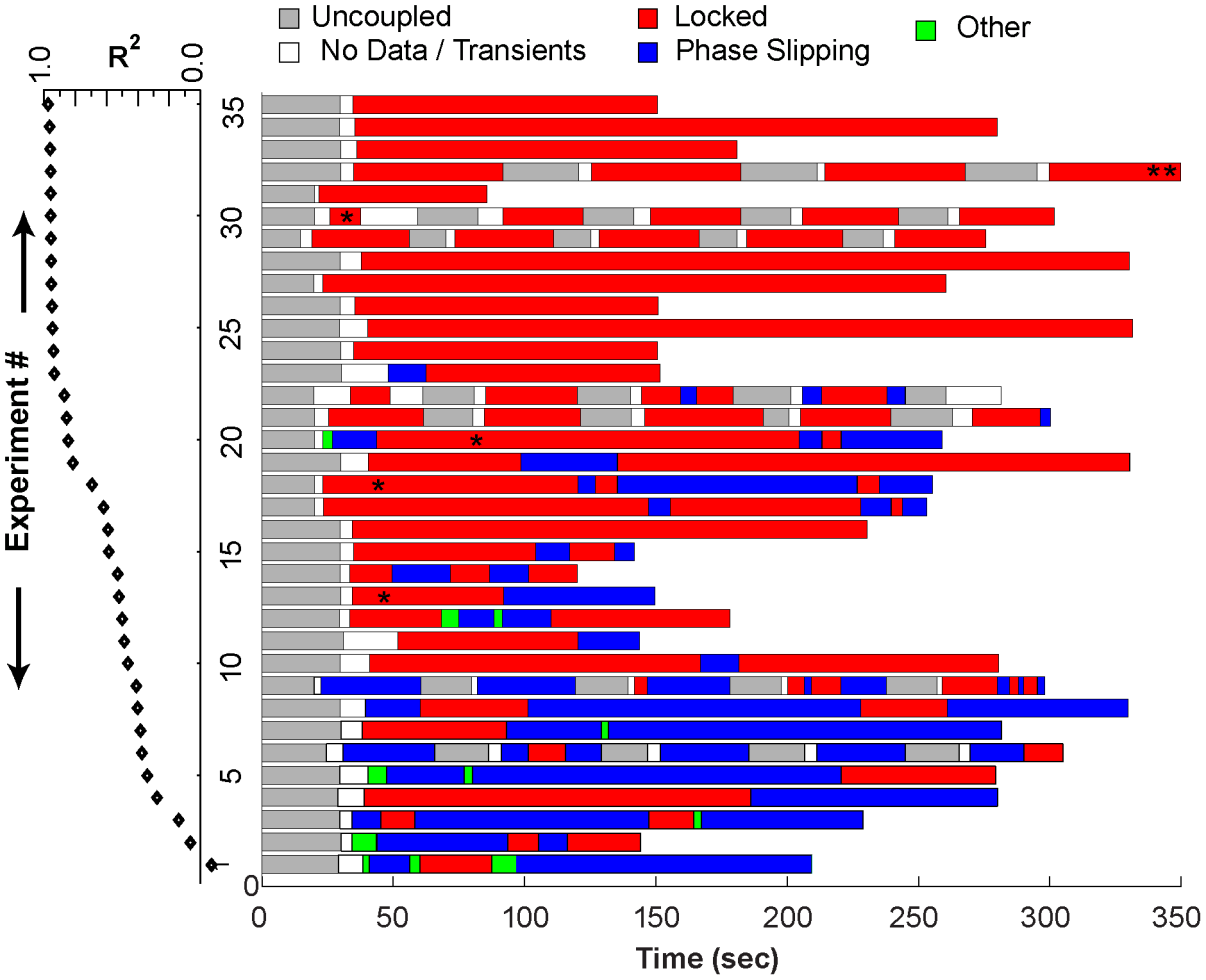
Schematic representation of network activity observed for 35 different hybrid circuits. Each experiment is represented by a color-coded band, indicating episodes of phase-locking (red), phase slipping (blue), no coupling (gray), initial transients or missing data (white) or unclassified (green). Red segments containing an asterisk were phase-locked with zero phase difference. Experiments were ordered with the lowest R^2^ statistic (black diamonds) during coupled episodes at the bottom. Data from experiment 32 (double asterisk) indicates that only the first 350 seconds of the record were shown, but the phase-locked pattern continued. The single asterisk within red segments corresponding to experiments 13, 18, 20 and 30 are episodes of near synchrony.

All 35 hybrid circuits showed episodes of phase locking, phase slipping, or both (see Figure 2). In Figure 3, the horizontal axis represents time and each experiment is represented on one row. The experiments are ranked vertically in order of R^2^, a metric of the consistency of phase locking during coupling. Coupled neurons with high R^2^ values remain phase-locked for the entire experiment duration. As R^2^ decreases, more episodes of phase slipping and undefined activity occur in the hybrid circuit. Note that an experiment with motif changes can nonetheless have a higher R^2^ value than one that is always phase-locked, particularly when the network phase in the first case has less variability than the network phase values in the second case. Well-defined network motifs occurred in every experiment.

### Dynamical motifs can be described using iterated pulse-coupled maps derived from PRCs

**Figure 4:**
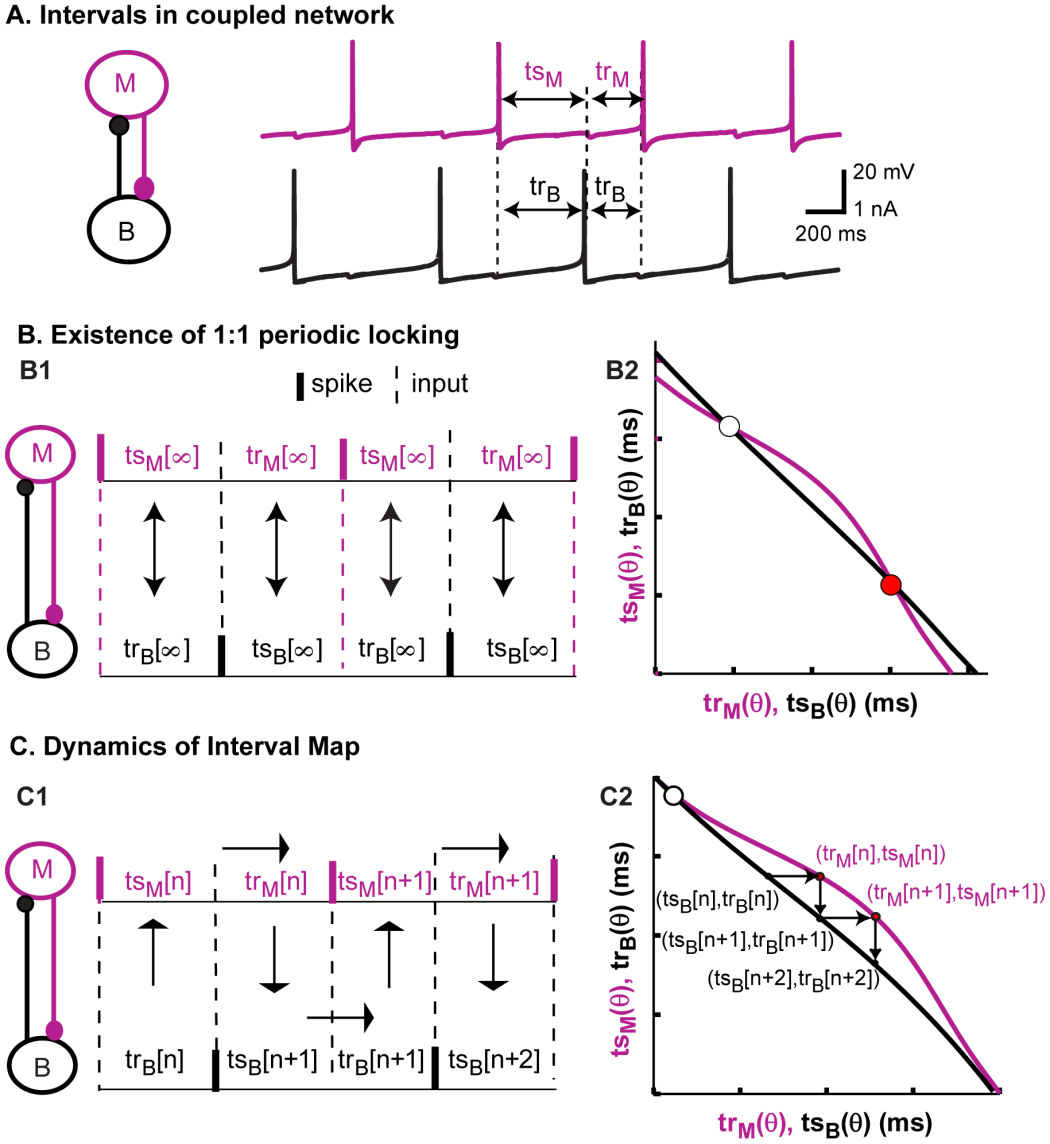
Predicting network activity using the PRC-based maps. (A) The stimulus and recovery intervals measured for the PRC can be used to predict the activity observed when the neurons are coupled; a spike in one neuron corresponds to an inhibitory input to the other neuron. (B1) In steady locked modes, the values of the intervals are fixed and the recovery interval in one neuron is equal to the stimulus interval in the other. (B2) Fixed points, or intersections of the curves on the *ts-tr* plane, can be used to predict steady one to one phase-locked modes. (C1) The recovery interval in one neuron is the next stimulus interval in the other neuron. The stimulus interval predicts the next recovery interval in the same neuron based on the PRC. (C2) Movement from curve to curve in the *ts-tr* plane corresponds to changes in network phase and sometimes in spiking order.

When two neurons are coupled, the dynamics of the resulting network can be predicted by plotting the PRC data of each neuron in the *ts-tr* plane. As stated in the Methods, the stimulus interval *ts* is the interval between the previous spike and an input from the other neuron, whereas the recovery interval *tr* is the interval between the arrival of an input and the next spike. We refer to these curves in the *ts-tr* plane as interaction curves. In contrast to the weak coupling approach [39], [40] using the infinitesimal PRC (iPRC), we do not ignore the effects of phase resetting on the network period nor do we require the relative phase of the neurons to change slowly compared to their absolute phases, however we do require that the coupling be pulsatile, meaning that the effects of an input die out quickly, before the next event occurs. In the coupled system, the stimulus interval for one neuron equals the recovery interval for the other neuron (Figure 4A) and vice versa. In a one-to-one periodic phase-locked mode, the intervals do not change from cycle to cycle, indicated by the index ∞ in Figure 4B1. For each neuron, a pair of stimulus and recovery intervals correspond to each phase at which an input is received (Figure 4A). In Figure 4B2, the stimulus interval for one neuron (magenta, model neuron) is plotted on the x-axis and the corresponding recovery interval is plotted on the y-axis, whereas the stimulus interval for the other neuron (black, biological neuron) is plotted on the y-axis and recovery interval on the x-axis. Therefore the two pairs of stimulus and recovery intervals (in two different neurons) that must be equal in a phase-locked mode are plotted on the same axes. The intersections of these curves then correspond to any possible periodic phase-locked modes of the two neuron network, as well as to fixed points of the *ts-tr* map in Figure 4C that is described below.

The information in the *ts-tr* interaction curves is not restricted to the location of the fixed points, but also provides the transient dynamics that may lead to a phase-locked mode or persist indefinitely in the absence of such a mode. The stimulus interval in one neuron determines the recovery interval in that same neuron; this leads to a map (Figure 4C1) with the following dynamics. The index *n* indicates successive cycles in the model neuron. The movement of the operating point from the black to the magenta curve is constrained to be horizontal because the recovery interval in the biological neuron determines the next stimulus interval in the model neuron (*tr_B_[n] = tr_M_[n]*). Similarly, the movement of the operating point from the magenta to the black curve is constrained to be vertical because the recovery interval in the model neuron determines the next stimulus interval in the biological neuron (*tr_M_[n] = tr_B_[n+1]*). For a stable fixed point that attracts nearby trajectories, the magenta curve with the coordinates listed in the order (*tr_M_,ts_M_*) curve must have a steeper slope ([41], see also derivation in Text S1) than the black curve in which the coordinates are listed in the opposite order (*ts_B_,tr_B_*), otherwise the point is unstable and repels trajectories. The white circle in Figure 4C2 (and B2) repels trajectories and therefore denotes an unstable fixed point, whereas the red circle in Figure 4B2 is stable because nearby trajectories would be attracted rather than repelled.

**Figure 5:**
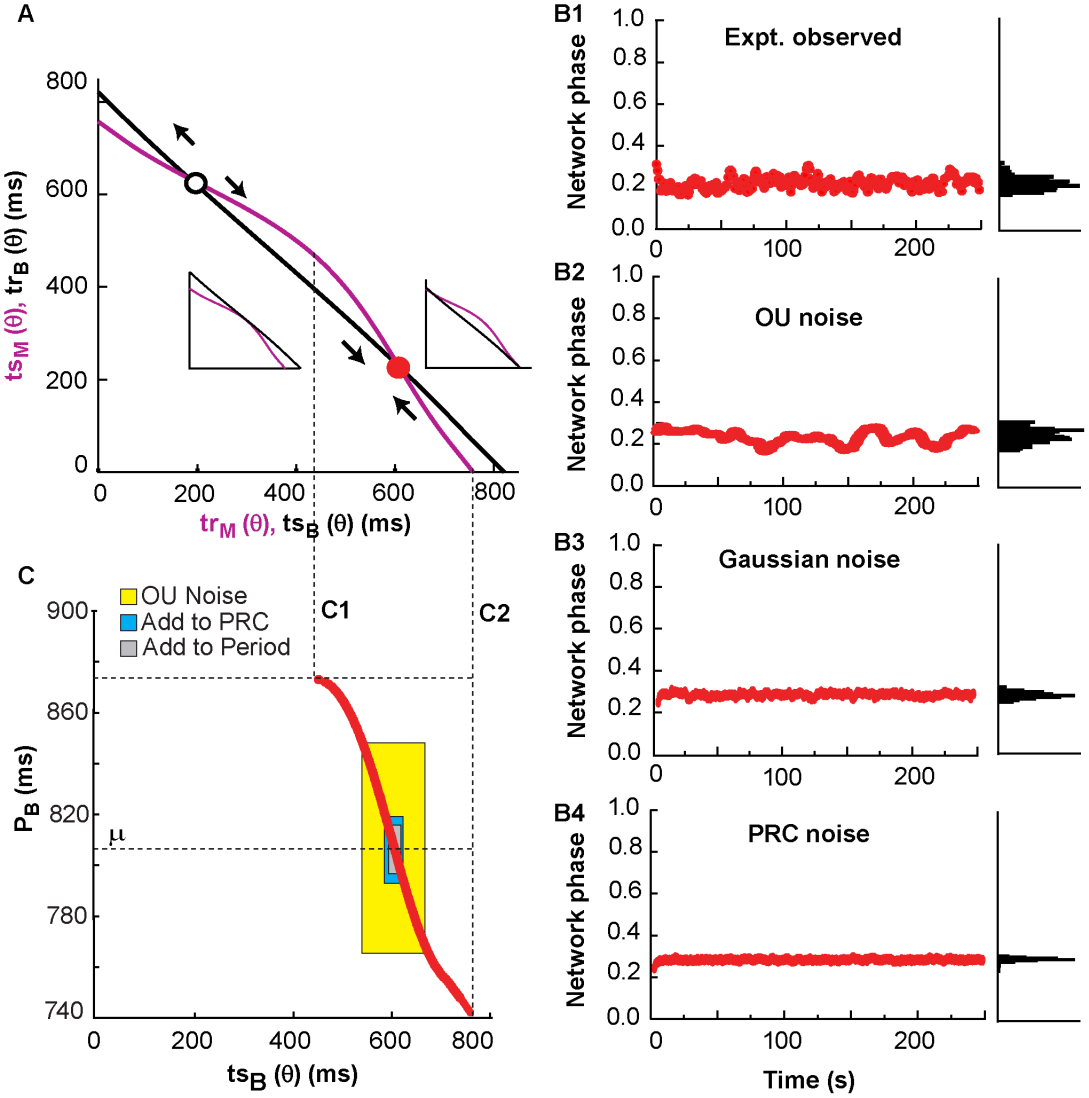
Scenario of robust phase-locking. Representative example of stationary phase-locking from experiment 34 (Table 1). (A) The interaction curves (obtained with mean period μ = 806.3 ms measured prior to the onset of coupling) intersect twice producing one stable (red) and one unstable fixed point (white). B. Histograms of network phase shown at right. (B1) Experimentally observed network phase, which is equal to *ts_M_/P_M_*, where *ts_M_* and *P_M_* are the stimulus and period of the model neuron. The phase remains locked (red circles) during the whole course of the experiment. (B2) Simulated network phase with Ornstein-Uhlenbeck period noise (σ = 0.1047 and τ = 80630). (B3) Simulated network phase with Gaussian noise (σ = 0.00532). (B4) Simulated network phase with Gaussian noise added to the PRC (σ = 0.00379). (C) Range of periods of the biological neuron in which the *ts-tr* curves have a stable intersection (red dots). For all the three forms of noise, (μ±2σ_eff_) remains inside in the region where the stable fixed point exists. Insets in A show how the stable intersection is lost at the dashed lines corresponsing to C1 (left inset) and C2 (right inset).

Figure 5 shows an example of stationary phase locking that occurs when there is a stable fixed point on the PRC-based map. The *ts-tr* interaction curves in Figure 5A were generated with the period observed in the biological neuron just prior to coupling, and intersect at two fixed points, one unstable (white) and one stable (red). The latter corresponds to the phase locking observed in both experiments and simulations. The insets reflect that a change in the intrinsic period of the biological neuron results in a shift of the *ts-tr* interaction curve for the biological neuron. As the neuron period gets longer, the curve shifts upward and rightward along the x-y diagonal (see left inset), and as the period gets shorter, the curve shifts inward toward the *ts-tr* origin (see right inset). The network phase remains relatively constant for the entire duration of coupling in this experiment (Figure 5B1), resulting in a histogram of the network phases with a distinct peak. In simulation, we can produce a similar time series of network phases and histogram in the presence of the three types of noise (Figure 5B2-B4), although only the OU noise produces a sufficiently broad peak. Figure 5C explains why the phase locking is robust to noise. The red curve shows the location of the stable fixed point in terms of *ts_B_* (and *tr_M_*) as a function of the period of the biological neurons shown on the y-axis. The initial value of period (used as μ in the noise models) is shown by the lowermost dashed horizontal line labeled μ. The initial value of *ts_B_* at the fixed point is about 600 ms as shown in Figure 5A. If the period of the biological neuron decreases, the curves no longer intersect below a period of about 740 ms and a *ts_B_* of about 755 ms (rightmost vertical dashed line labeled C2) corresponding to the situation in the inset at right. Similarly, if the period of the biological neuron increases, the curves no longer intersect above a period of about 875 ms, and a *ts_B_* of about 430 ms (leftmost vertical dashed line labeled C1) corresponding to the situation in the inset at left. The *ts-tr* interaction curves are a snapshot of the constraints on the trajectories based on the current value of the period of the biological neuron. The variability in all three models constrains the 95% confidence interval of the intrinsic period (μ±2σ_eff_) to lie well within the range of periods that supports phase locking, and therefore constrains the variability in the network phase observed in Figure 5B2-4, and presumably in Figure 5B1 as well.

**Figure 6:**
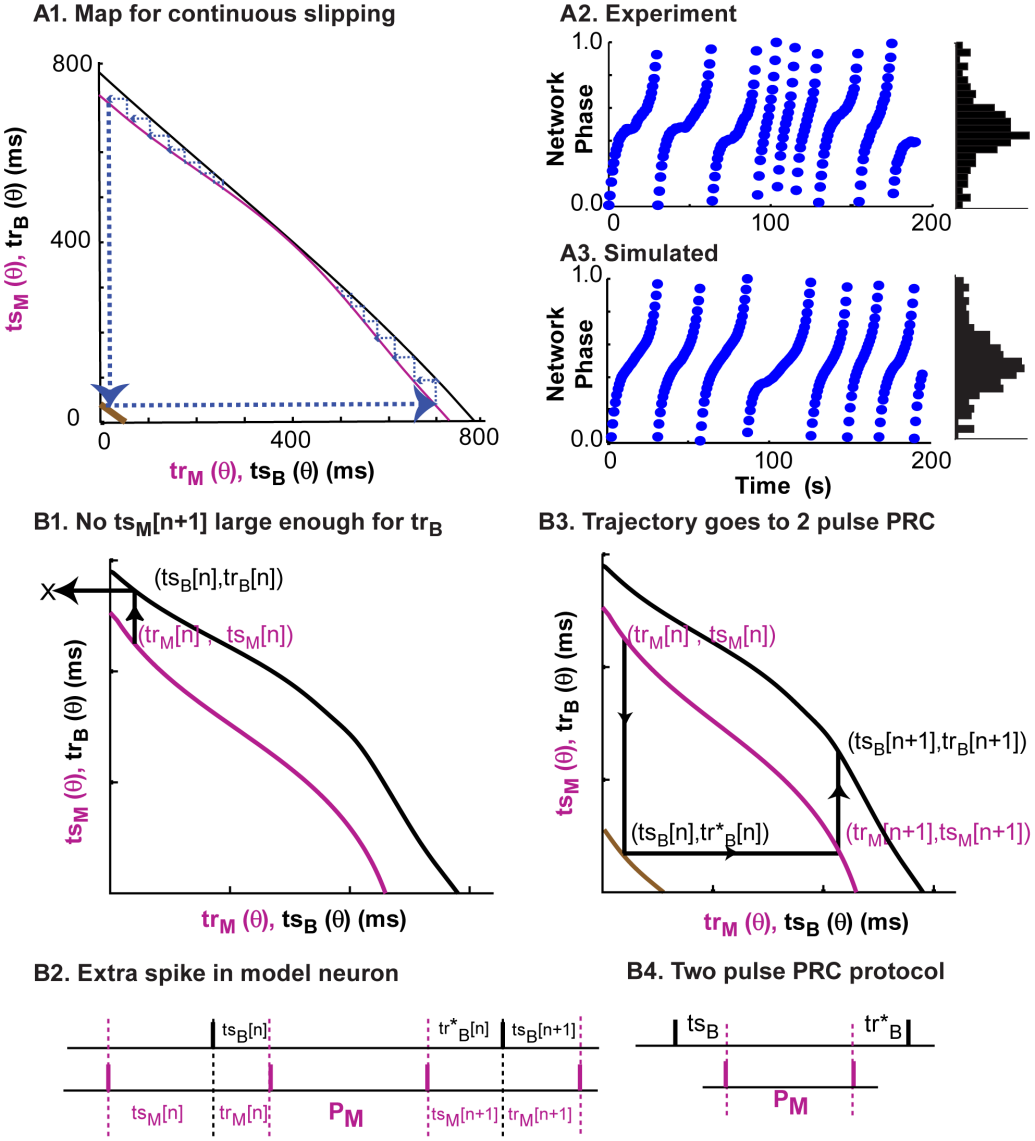
Phase slipping without “preferred” phase. (A) Representative example of phase slipping from experiment 5 (Table 1). (A1) The interaction curves do not intersect, and there is no fixed point in the system. The dashed blue lines show the sequence of intervals during the phase slips. Brown curve is the two pulse PRC explained in B. (A2) Experimentally observed network phase showing multiple slips in blue, resulting in a broad distribution of network phases in the histogram on the right that nonetheless has a clear peak. (A3) Simulated times series of network phases and histogram using the noisy map based on the interaction curves in B1, with Ornstein-Uhlenbeck noise (σ = 0.04225 and τ = 76790). The peak in the histogram corresponds to the closest point of approach of the curves. (B) Schematic illustrating how one neuron spikes again before recovery interval in partner ends. (B1) Hypothetical interaction curves show an example of a *tr_B_[n]* value that is larger than any value of *ts_M_[n]*, so the X on a dashed black line indicates a recovery interval that was too long to be physically realized. (B2) The spike pattern corresponding to B1 contains two successive spikes in the model neuron with no intervening spike in the biological neuron. (B3) The recovery interval tr*_B_[n] is obtained from *ts_B_[n]* by accounting for the two consecutive spikes in the partner using the brown interaction curve instead of the black one. The sequence of intervals shown on the map corresponds exactly to the firing pattern in B2. (B4) In order to determine tr*_B_[n], two inputs separated by the intrinsic period in the partner are applied.

The PRC-based map helps illustrate what happens during phase slipping. Figure 6A1 shows the *ts-tr* interaction curves using the period observed in the biological neuron just prior to coupling, with a trajectory around the PRC-based map indicated by dashed blue lines with arrows indicating direction. Every time one neuron spikes, a vertical or horizontal “step” is taken between the two curves. The trajectory spends more time near the point of closest approach between the two curves because it takes smaller steps in that region. Here, the ghost of a fixed point that exists at a slightly different set of parameters (at which the curves do intersect) has a significant impact on the dynamics [42], [43]. Figure 6A2 shows the sequence of network phases observed during the long episode of slipping in experiment 5, and the histogram at right shows a broad peak in the network phases. The peak and distribution of the histogram of the network phases produced by a map based on the PRC with OU noise in the period and shown in Figure 6A3 was in reasonable agreement with the experimental data in Figure 6A2. The peak of the histogram is due to the tendency to stick near a phase corresponding to the point of closest approach of the curves in Figure 6A1.

Each phase slip in the network activity is associated with a trajectory that dropped down from the upper left edge of the map and was reinjected at the lower right edge (Figure 6A1). Figure 6B displays the mechanism for dropping off the map at the upper left and returning at the lower left. This occurs when the next recovery interval in one neuron (the biological neuron in Figure 6B1) is so long that the other neuron (the model neuron in Figure 6B2) spikes twice during one biological neuron period. We defined a recovery interval tr* (see two pulse PRC protocol in Figure 6B4) that gives the interval to the next spike after two inputs separated by the intrinsic frequency of the partner neuron are received (brown curve in Figure 6B3). Therefore the trajectory is reinjected at the lower right when it falls off the upper left, and vice versa. The recovery interval tr* was not measured, but instead was calculated from the previously collected phase resetting data by assuming that the second input was received at a phase determined not only by the elapsed time but by taking into account the phase resetting from the first pulse. The only way to transition between the ends of the map is for one neuron to spike twice in a row, and the modified map can handle any firing pattern in which any single neuron does not spike more than twice in a row. Across a phase slip transition, there is a change [44] in leader-follower pattern of the neurons. It is important to note that there exists a similar analogy of ghost attractor and cycle slipping in return map of Poincare phase map of neural oscillators [45].

### The dynamics of motifs are variable, and switching between motifs is possible

Phase locking and phase slipping do not always persist throughout an experiment; as seen in Figure 3, motifs can vary over time. Of the 35 hybrid experiments presented here, 12 experiments represent the case where the system is phase-locked (Figure 3, experiment 16 and experiments 24–35). In the remaining 23 cases, the coupled neurons transition between phase locking, phase slipping, and undefined phase relationships (Figure 3, experiments 1–15 and experiments 17–23). It is likely these transitions are due to fluctuations in the intrinsic spiking frequency of the biological neuron. As illustrated in Figure 5A, shifts in the *ts-tr* interaction curves due to drift in the period of the biological neuron can move, create, or eliminate the fixed points of the system.

**Figure 7:**
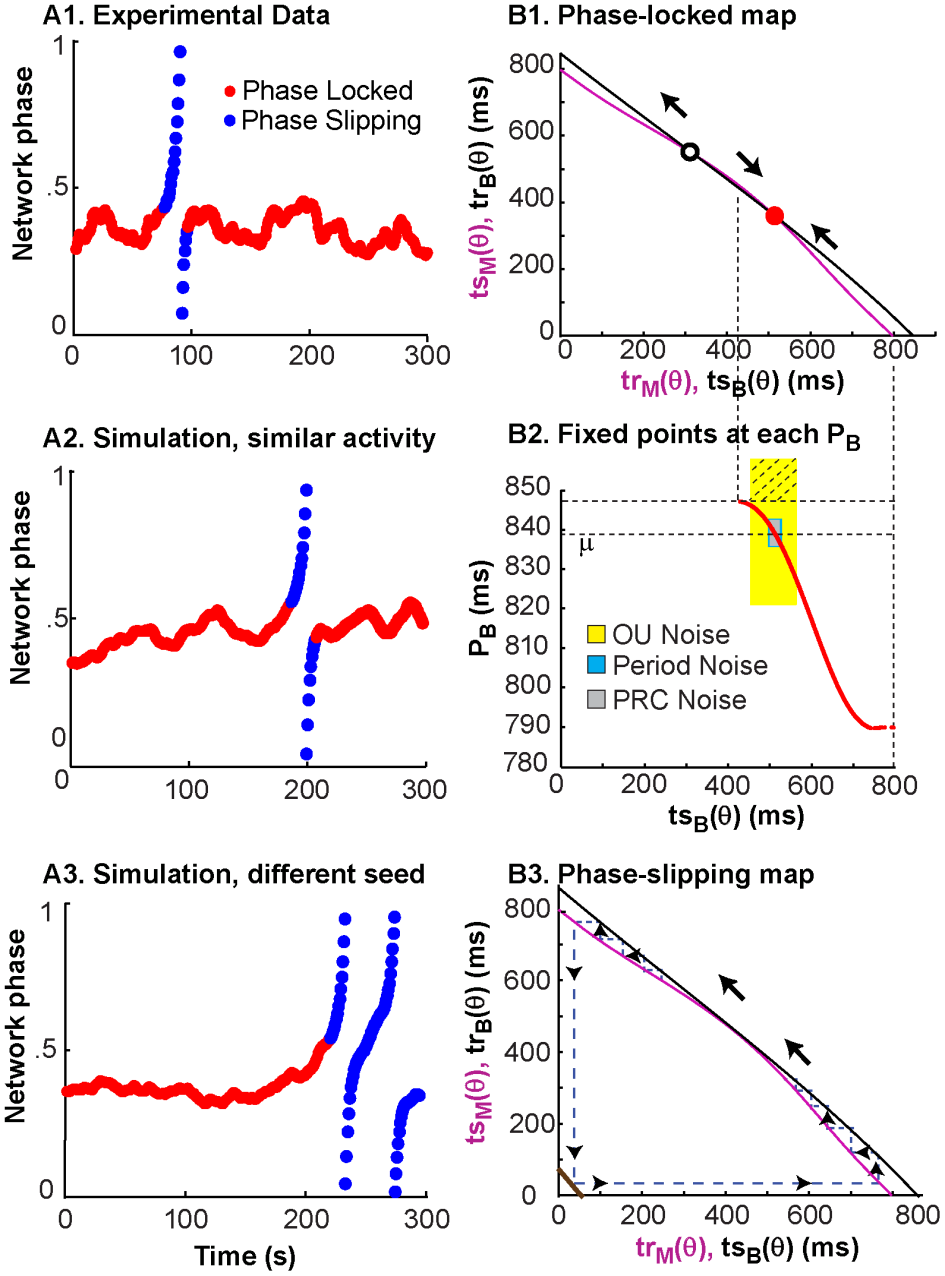
Representative example of nonstationary activity corresponding to nonrobust locking and drift-induced bifurcations. (A1) Observed network phase shows phase-locking (red) with a single phase slipping episode (blue) from experiment 19. (A2) Simulation of the network phase with Ornstein-Uhlenbeck (OU) noise (σ = 0.0453 and τ = 83882) showing a transition from phase locking to phase-slipping and vice-versa. (A3) For the case of OU noise, the simulated network phase shows sensitivity to different initial random seeds, and it can produce qualitatively different results. (B1) The *ts-tr* curves using the period of the biological neuron at the start of the experiment have one unstable (white dot) and one stable (red dot) intersection, corresponding to the phase-locked mode observed at the beginning of experiments and simulations. (B2) The two horizontal dashed lines give the range of the period of biological neuron where a stable fixed point exists. The 95% confidence region of the period (μ±2σ_eff_) contains values outside the phase locking regime (crosshatched region of yellow rectangle only for the Ornstein-Uhlenbeck noise model). In this region, intersection of the *ts-tr* curves are lost. (B3) As the biological neuron period drifts, the interaction curve for the biological neuron moves relative to that for the model neuron, with a value of 838.8 ms in B1 compared to 850.7 ms in B3. Slips are caused when the two fixed points in B1 collide, leaving no intersection as in B3. The two fixed points may re-emerge resulting in renewed locking.

**Figure 8:**
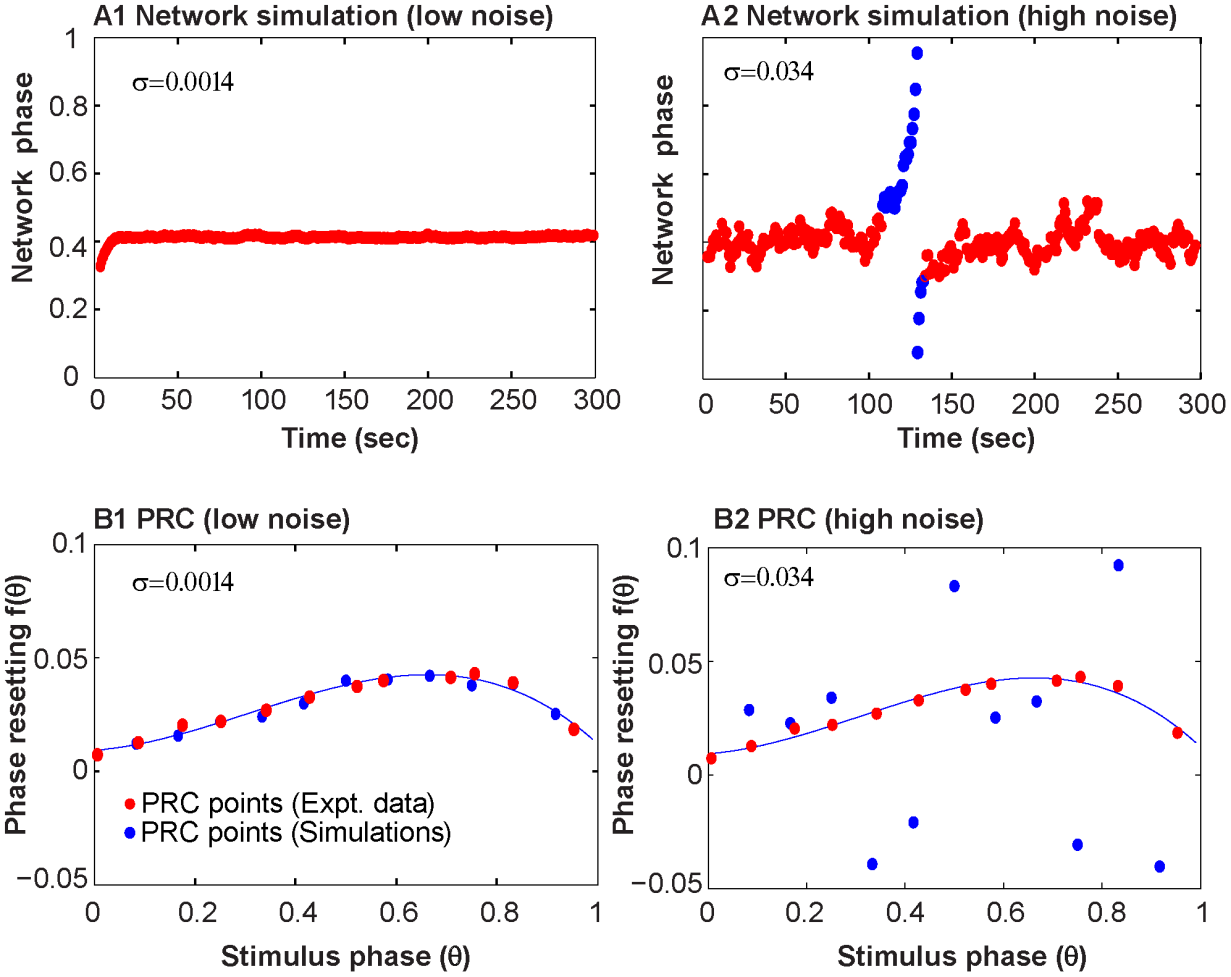
Gaussian noise added to PRC cannot mimic both the hybrid circuit data and the noise level in the PRC with the same parameter value. A. Simulation of hybrid network for Experiment 19 illustrated in Figure 7A1. (A1) Low noise case does not capture network dynamics. (A2) High noise case better reproduces network dynamics. (B). Comparison of experimental (red dots) and representative simulated (blue dots) PRC measurements. The experimentally measured PRC from the biological cell in experiment 19 is fitted with a 3^rd^ degree polynomial (black curve). (B1) The low noise case captures the variability in the PRC quite well. (B2) The high noise case vastly overestimates variability in PRC. For low noise σ = 0.0014 and for high noise σ = 0.0344.

Figure 7A1 shows experimentally observed phase-locked activity with a single slip. One simulated coupling experiment (Figure 7A2) with the Ornstein-Uhlenbeck noise process mimics the single slip from phase-locked activity observed in the experiment. However, the simulations are sensitive to initial conditions, and using a different random seed for the noise produces a different pattern of transitions (Figure 7A3). The *ts-tr* interaction curves coupled with the effective standard deviation of the period for the various noise processes, can explain why the OU process, but not the other noise models, was able to mimic the transition to slipping activity. The red curve in Figure 7B2 again shows how the location of the stable fixed point in terms of *ts_B_* (and *tr_M_*) change and disappear as the period is increased or decreased. The initial value of period (used as μ in the noise models) is shown by the lowermost dashed horizontal line in Figure 7B2, and was used to generate the *ts-tr* interaction curves shown in Figure 7B1 that have two intersections, including a stable fixed point that predicts the initially observed phase locking. However, if the period of the biological neuron increases from the initial value of 839 ms above 845 ms, the stable intersection is lost, resulting in the *ts-tr* interaction curve shown in Figure 7B3 that produces phase slipping. The shaded regions in Figure 7B2 show that the OU noise model produces a larger standard deviation of the period, such that the 95% confidence interval of the intrinsic period (μ±2σ_eff_) includes periods that correspond to phase slipping (crosshatched region). On the other hand, the 95% confidence intervals for the other two models lie well within the region of periods corresponding to phase locking, so slipping was never observed for any random seed in simulations of this particular experiment using those noise models. Figure 8 gives an example in which the same experiment (number 19) illustrated in Figure 7 was simulated with Gaussian noise added to the phase resetting. In this case, as in the case in which Gaussian noise is added to the period, there is no dependence of the noise in one cycle on that in the previous cycle. Figure 8A illustrates a typical fit to the network dynamics with low (panel A1) and high (panel A2) noise levels. The high noise levels are able to capture the network dynamics, but the low noise level fails. Conversely, Figure 8B shows that the low noise level (panel B1) faithfully captures the low variability in the PRC as measured, but the high noise level greatly overestimates the variability. Figure S1 shows that the simulations of this experiment with Gaussian noise added to the period fails in exactly the same way.

**Figure 9:**
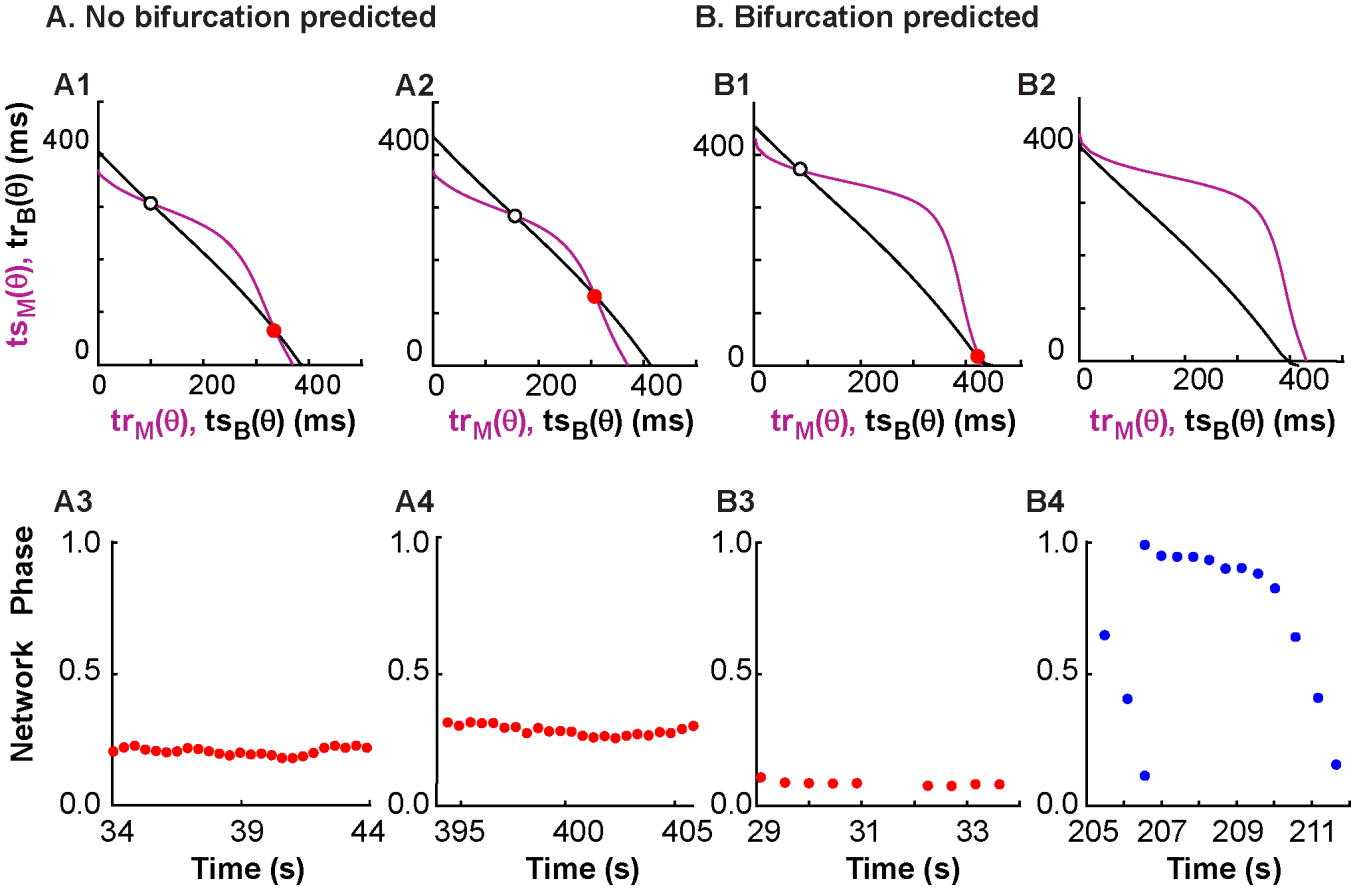
Prediction of the robustness of network activity to period noise. Coupling between the neurons was turned off between panels, the period of the isolated biological neuron was re-measured, and the prediction of network activity was adjusted based on the newly measured period by shifting the black curve representing the PRC information for the biological neuron. (A) Activity from experiment 32 was predicted to be robust to period noise. The prediction does not change much between A1, based on the period of 396.3 ms before the first coupling episode, and A2, based on the period of 425.56 ms before the last coupling episode. The observed activity in A3 (first episode) and A4 (last episode) is consistently phase-locked with no bifurcations. (B) Activity from experiment 22 is not predicted to be robust to period noise, with the interaction curves shown in B1and B2 reflecting the average periods in the biological neuron of 440.2 and 405 ms prior to the first and last coupled episodes respectively. In B1, the red dot predicts stable locking at a nonzero phase as observed in the first episode in B3. The period shift in the biological neuron causes all intersections to disappear in B2, resulting in the phase slipping in the last episode (B4).

Robustness to shifts in period on the PRC-based map translates to robustness of phase-locked network activity. This robustness to period changes is represented by the shape and proximity of curves on the *ts-tr* plane. The network phase of a system with one curvy *ts-tr* neuron representation and one straight *ts-tr* neuron representation is more likely to stay phase-locked than a system where both neuron representations are very straight; this robustness also depends on the position of one curve with respect to the other, since intersections near the edges of *ts-tr* curves will be susceptible to noise-induced bifurcations. Figure 1 showed that increasing the coupling strength increases the curvature of the interaction curves for the models (and experiments, not shown) used in this study. In Figure 9, the coupling between neurons was turned off between panels, and a snapshot of the *ts-tr* interaction curves (top row) was generated for each coupled episode; the black curve representing the biological neuron was shifted according to the average period measured during the previous uncoupled episode. Figure 9A shows a network with stable phase locking; a change in biological neuron period did not disrupt the motif. Figure 9B shows a case where network activity transitions from locked to slipping, due to the decreasing biological neuron period and the resulting loss of the stable fixed point.

### Motif variability can be described by a model that accounts for slow noise in the intrinsic variability of the oscillations

One advantage of using a hybrid circuit model is that only the biological neuron contributes noise to the system. We simulated two coupled neurons using iterated maps derived from the PRCs measured from the biological and model neurons during experiments. The model neuron was noiseless and had a constant intrinsic period. Three types of noise models were used in only the simulated biological neuron to consider different types of biological variability. Gaussian noise added to the simulated biological PRC mimics the approach in [15], [34] and describes uncertainty in the PRC itself. Gaussian noise added to the simulated biological neuron period represents uncertainty in the measurement of spikes as well as intrinsic variability in the timing of neuronal spikes. Modeling the period of the biological neuron as an OU process captures intrinsic variability in spike timing in the biological neuron and measurement errors, as well as slow, long-term trends in the intrinsic period.

**Figure 10:**
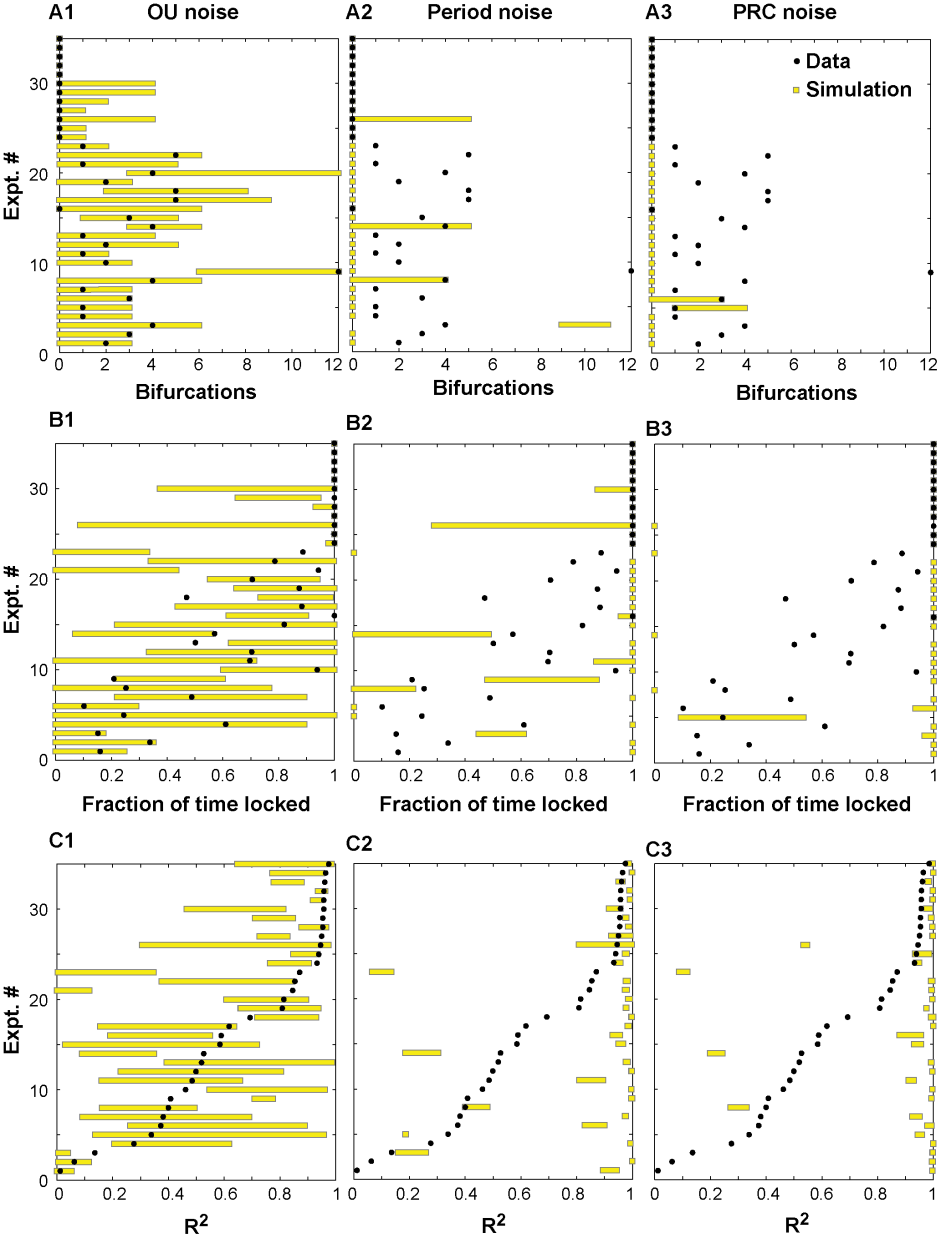
Performance metrics for noise models. The metrics are (A) Bifurcation, or the number of transitions between phase-locked and phase-slipping states, observed in each hybrid experiment; (B) Fraction of time in each experiment spent in a phase-locked state; and (C) the R^2^ metric for each experiment. In each panel, the y axis indicates an experiment number. For each experiment, the black dot represents the metric for that experiment, and the yellow bar represents the range of results for 10 simulations of each of the three noise models using 10 random seeds. Ideally, the bar (range of simulation results) should enclose the dot. The Ornstein-Uhlenbeck noise model in A1 brackets the experimental data in all cases, whereas Gaussian noise added to the period in A2 and PRC in A3 fail to bracket the observed bifurcation in most cases. (B) The Gaussian noise models in B2 and B3 had less effective variability than the OU model in B1, so the OU model was better able to capture fraction of time spent phase-locked. In contrast, the Gaussian noise models had a tendency to produce simulations that were always phase-locked, or to a lesser degree that were always slipping. (C) The OU model in C1 usually bracketed the data points for the R^2^ metric, but the other models (C2 and C3) in general did not.

Figure 10 shows the performance of the three noise models on three metrics: transitions between motifs (Figure 10A), the fraction of time spent phase-locked (Figure 10B), and the circular statistic R^2^ (Figure 10C). Experiments 24–35 spent 100% of the time phase-locked (see Figure 3 and Table 1) with no transitions, therefore their R^2^ is quite high. The vast majority of the time that the circuit was not phase-locked was spent slipping, so the performance on the metric of fraction of time spent slipping was similar and is not shown. The metrics are presented in terms of the range of values obtained for ten different simulations for each noise model for each experiment. In each of the ten simulations, the noise model was initialized with a different seed, and the pulse coupled network simulator was run for the same length of elapsed time as the original experiment. The parameters of the noise models for each experiment are given in Table 2. Since some simulations were quite sensitive to initial conditions, the best possible match would be that the values of the metrics obtained in the ten simulations bracket the value actually observed in the experiment. The OU model was calibrated to fit the bifurcation data in Figure 10A, and all experimental data points (black dots) are bracketed by the range of simulation results (yellow bars) for that model. Note that the fits given for the OU model in Table 2 are not unique (see Supplementary Figure S2), however, two consistent trends emerged. Decreasing the time constant τ for mean reversion somewhat reduced the PRC noise and the numbers of bifurcations introduced by increasing the noise intensity σ, and the variability between runs was greater for larger mean reversion time constants. The other models did a poor job of capturing the bifurcations, or transitions between motifs. In general, the transitions identified in those models were sticky regimes (meaning they exhibited a “preferred” phase) during phase slipping that the algorithm identified as phase locking episodes (see Supplementary Figure S3), and they failed to capture many transitions, such as the one illustrated in Figure 8 (see also Supplementary Figure S1). Most black data points that represent a nonzero number of bifurcations were not bracketed by the simulations for the two Gaussian models because they failed to exhibit history dependence of the period (see Supplementary Figure S4). Although the OU model was not calibrated to capture the fraction of time spent phase-locked data and R^2^, the OU model clearly outperformed the other models on these metrics as well. The Gaussian noise models had less effective variability in network activity than the OU model, so the OU model was better able to capture fraction of time spent phase-locked. In contrast, the Gaussian noise models had a tendency to produce simulations that were always phase-locked, or to a lesser degree that were always slipping. The OU models usually bracketed the data points for the R^2^ metric, but the other models in general did not.

## Discussion

### Dynamics in hybrid coupled networks are captured in PRC-based maps

We have shown here that multiple types of network activity occur in hybrid circuits of one biological and one computational neuron. We used PRC-based maps to explain activity observed in hybrid circuits of one biological and one computational model neuron. These maps are based on interaction curves that give a snapshot in time of the dynamics expected, assuming that the intrinsic periods of the neurons remain relatively constant during the time window in question. The fixed points, or equilibria, of the PRC-based maps presented here are given by the intersections of the *ts-tr* interaction curves for the two neurons, and correspond to one-to-one phase-locked modes in the circuit. However, perturbations from these fixed points are inevitable in a noisy system, and the nonequilibrium dynamics of the map as trajectories flow between the interaction curves gives the system dynamics for perturbations away from fixed points, and also in the complete absence of fixed points. The two most common dynamical motifs, phase slipping and phase locking, can occur under variable circumstances. The existence of a stable fixed point predicts phase locking, and the absence of a stable fixed point predicts continuous phase slipping. However, the interaction curves themselves can change over time because they are based on the intrinsic period of the component neurons. As the period of the biological neuron slows down, the PRC-based interaction curve for that neuron moves outward; as it speeds up, the curve moves in toward the origin. This motion can change where the curves intersect, effectively changing where the fixed points are located. This can result in a shift of the network phase if the system remains phase-locked, or a transition to phase slipping if the stable fixed point is lost.

### The nature of noise in neural circuits

The synaptic component of the noise is thought to be dominant compared to intrinsic noise sources, and noise is often modeled as a high conductance state [12] in which both the inhibitory and excitatory conductances are Ornstein-Uhlenbeck processes. Noise in neural systems [11] is also often modeled as a random walk in the membrane potential due to excitatory and inhibitory synaptic currents whose interevent times are generated by a Poisson process; the membrane potential is continuously pulled back toward the resting potential with a characteristic time constant. This approach may be appropriate for normally quiescent neurons, but additional considerations may apply for oscillatory neurons. An oscillatory neuron is not merely pulled towards a resting potential, but instead has a characteristic cycle period determined by the inverse of the η_i_ term in Eq. 1. Under our interpretation, intrinsic membrane noise in an oscillator can be modeled as perturbing the intrinsic cycle period, or 1/η_i_. The stochastic form of equation 1 is referred to as a Langevin phase equation; to our knowledge we are the first to model the cycle period itself as an Ornstein-Uhlenbeck random process.

Here, we show that normally distributed noise added to cause jitter in either the PRC or the period was insufficient to capture the dynamics of the observed switches between motifs. Instead, history-dependence (see Supplementary Figure S4), presumably mediated by stochastic processes with slow dynamics that allowed the fast jitter to accumulate over time, was required in our simulations in order to replicate our experimental observations of hybrid circuit dynamics. The period of the biological neuron was modeled as an Ornstein-Uhlenbeck (OU) process with the mean reversion modeled as being on the order of 10–1000 cycle periods. The mean reversion was included because it is quite likely that period of biological oscillators is homeostatically regulated within a physiologically relevant range, but this term was not crucial (see Supplementary Figure S4) or particularly well-characterized in our data. Support for treating the period as a random process is provided by observations of slow fluctuations in oscillatory period when neurons that are nonoscillatory in a slice preparation, such as stellate cells in entorhinal cortex and CA1 hippocampal OLM cells, are made to oscillate using current injection [16], and by the successful use of an OU model to characterize the variability in period in CA1 pyramidal neurons under similar conditions [46]. There is strong support for the idea that neuronal circuits possess both intrinsic and synaptic mechanisms that operate over hours to days to maintain firing around a homeostatic stable point [47]. However, these studies were not focused on the homeostatic regulation of the intrinsic firing rate of oscillating neurons on a time scale of tens of seconds to minutes as we suggest here. The underlying biophysical mechanisms that could produce an OU process in period (or alternatively in frequency) in physiological neural oscillators are not clear. However, Schwalger [20] and Fisch et al. [48] have shown that stochastic slow ionic currents may be well-represented by OU noise in neurons.

Typically noise in the Langevin equation for phase is formulated as an additive term to the frequency [49], [50] or to the phase resetting [15], [16], [34], [51], [52] or both [53]. Another method is to convolve the noise with the infinitesimal phase resetting curve (iPRC) and then add to the phase [54] or frequency [55]. It is unlikely any of these methods could capture the transitions between modes observed in our experiments because they lack the crucial history dependence of the period from one cycle to the next. If the period of oscillatory neurons in general can be modeled as a history-dependent and likely mean-reverting random process, theoretical results to date on the behavior of ensembles of noisy biological oscillators may require re-evaluation and modification. Interestingly, theta oscillations in the local field potential of the hippocampus and prefrontal cortex also show a pattern of small frequency fluctuations over time, referred to as the *microstructure* of the theta rhythm [56], so the concept of the period as a random process may be extendable to network oscillations as well.

### Robustness in noisy circuits

The predictions of phase locking under weak coupling assumptions [39], [40] are independent of coupling strength (as long as it is weak) for noisy identical oscillators, but clearly a minimum coupling strength is required to overcome ubiquitous biological noise. Effects of heterogeneity in period have usually been studied [26], [57], [58] assuming that the intrinsic period for each neuron is relatively constant. We examined the consequences of fluctuations in the period of neuronal oscillators in the absence of additional synaptic input using the dynamic clamp. In our hybrid circuit experiments, the biological synaptic inputs were silenced using high Mg^2+^, low Ca ^2+^ solution [25]; we found that the assumption of a slow variation in the period of the oscillatory biological neurons, even in the absence of synaptic input, produces the best fit to our data. We show explicitly the relationship between the level of variability and the tendency to remain phase-locked, as well as the effect of coupling strength on stabilizing phase locking. In our hybrid circuits, the robustness of network phase locking was related to the degree of curvature of the two interaction curves that generate the PRC-based map, as well as the amount of spatial separation between the two curves. The degree of heterogeneity in frequency largely determines the spatial separation, or difference in the x and y axis crossing points of the interaction curves. The interaction curves in Figures 5A and 7B1 have similar separation on the x and y axes, but the greater curvature of the magenta curves in Fig 5A clearly means that greater separation can be tolerated before the intersections between the interaction curves are lost. Curvature is enhanced by strengthening the synaptic coupling, and spatial separation increases with increasing heterogeneity in the intrinsic spiking frequencies. These two factors determine the amount of effective variability that can be tolerated without disrupting the locking (see Figure 5 and 7). Other investigators [16] have responded to the variability in period by utilizing a controller to stabilize the intrinsic period in order to test the predictions of weak coupling, which presumes that the coupled period is equal or very nearly equal to the intrinsic period. The direct effect of slow trends in the period of component neural oscillators on network activity has not been previously investigated. This slow form of intrinsic noise may have important implications for synchronization in neuronal networks.

### Generalization to larger networks

These results are mainly of interest for their implications for larger networks, such as central pattern generating networks and hippocampal and cortical networks that subserve cognitive functions. There are two immediately apparent ways to generalize these results to larger networks. One is to generalize [36], [59], [60] from two neurons to two subpopulations of neurons in which the neurons in each subpopulation are different from those in the other subpopulation, but relatively homogeneous within a population. Homogeneity in frequency might be enforced by electrical coupling within but not between subpopulations for example. Another method is to directly scale up to larger networks; in this case our contribution is to suggest that slow intrinsic noise in the period that has not previously been considered may play a role in the collective dynamics of networks of coupled oscillators.

## Supporting Information

Figure S1 **Gaussian noise added to the period cannot mimic both the hybrid circuit data and the noise level in the PRC with the same parameter value.** A. Network phase data replotted from Figure 7A for experiment 19. B. Time course of the unobservable intrinsic period of the biological neuron during simulations of this experiment for σ = 0.00213 (blue trace) and σ = 0.03 (red trace). The center dashed line shows the initial (and mean period) whereas the solid horizontal lines indicate the values of the period between which an intersection exists in the *ts-tr* curves (see Figure 7b). The blue trace with low noise obscures the dashed line for mean period. C. Simulation of hybrid network for low noise (C1) and high noise (C2) case. D. Comparison of experimental (red dots) and representative simulated (blue dots) PRC measurements with low noise (D1) and high noise (D2).
(PDF)

Figure S2 **A parameter regime spanning a wide range of τ but a narrow range of σ fits both the PRC and hybrid circuit data for the OU model.** The parameter grid was sampled at the red dots, and the parenthetical expressions indicate the squared error ratio for the simulated to experimental PRCs as well as the range of bifurcations that were observed in ten random simulations of the hybrid circuit. The blue ellipses indicate the parameter space where the ratio is near 1 and the bifurcation range includes zero, since these experiments were always phase locked. (A) Experiment 25, (B) Experiment 27, (C) Experiment 28, (D) Experiment 34.
(PDF)

Figure S3 **Added Gaussian noise only picks up easily accessible, and sometimes questionable, bifurcations.** A. Network phase of hybrid circuit for Experiment 14 with phase slipping (blue dots) punctuated by “sticky” phase locking (red dots). B. Simulations confirm brief episodes identified as phase locked. C. Interaction (*ts*-*tr*) curves just miss intersecting, so a small amount of change in the biological period can cause an intersection (and phase locking) to occur. D. Time course of the unobservable intrinsic period of the biological neuron during simulations of this experiment (blue trace). The top dashed line shows the initial (and mean period) whereas the solid horizontal lines indicate the values of the period between which an intersection exists in the *ts*-*tr* curves.
(PDF)

Figure S4 **History dependence of the period without mean reversion is sufficient to mimic both the hybrid circuit data and the noise level in the PRC with the same parameter value.** A. Network phase data replotted from Fig. 8A for experiment 19. B. Time course of the unobservable intrinsic period of the biological neuron during simulations of this experiment for σ = 0.0123 (magenta trace), σ = 0.0453 (blue trace) and σ = 0.3453 (red trace). The simulations used Eq. 2 without the term containing τ, so τ was effectively set to infinity.The center dashed line shows the initial period (but not the mean in this case), whereas the solid horizontal lines indicate the values of the period between which an intersection exists in the *ts*-*tr* curves (see Figure 7b). C. Autocorrelation values for the same three σ values as in B. D. Simulation of hybrid network for low noise (D1), medium noise (D2) and high noise (D3) case. E. Comparison of experimental (red dots) and representative simulated (blue dots) PRC measurements with low noise (E1) and medium noise (E2) and high noise (E3).
(PDF)

Text S1 **This supplementary material contains 1) a detailed explanation of the criteria for identifying phase slipping and phase-locked episodes, 2) a derivation of stability for the PRC-based map, and 3) a derivation of effective standard deviation of the period for noise added to the PRC.**
(PDF)
