# Supplementary material for: Slow Noise in the Period of a Biological Oscillator Underlies Gradual Trends and Abrupt Transitions in Phasic Relationships in Hybrid Neural Networks
Source: PLoS Comput Biol. 2014 May 15;10(5):e1003622. doi: 10.1371/journal.pcbi.1003622 (PMC4022488; doi:10.1371/journal.pcbi.1003622)
Supplement: Figure S1 — Gaussian noise added to the period cannot mimic both the hybrid circuit data and the noise level in the PRC with the same parameter value. A. Network phase data replotted from Figure 7A for experiment 19. B. Time course of the unobservable intrinsic period of the biological neuron during simulations of this experiment for σ = 0.00213 (blue trace) and σ = 0.03 (red trace). The center dashed line shows the initial (and mean period) whereas the solid horizontal lines indicate the values of the period between which an intersection exists in the ts-tr curves (see Figure 7b). The blue trace with low noise obscures the dashed line for mean period. C. Simulation of hybrid network for low noise (C1) and high noise (C2) case. D. Comparison of experimental (red dots) and representative simulated (blue dots) PRC measurements with low noise (D1) and high noise (D2). (PDF) [file pcbi.1003622.s001.pdf]

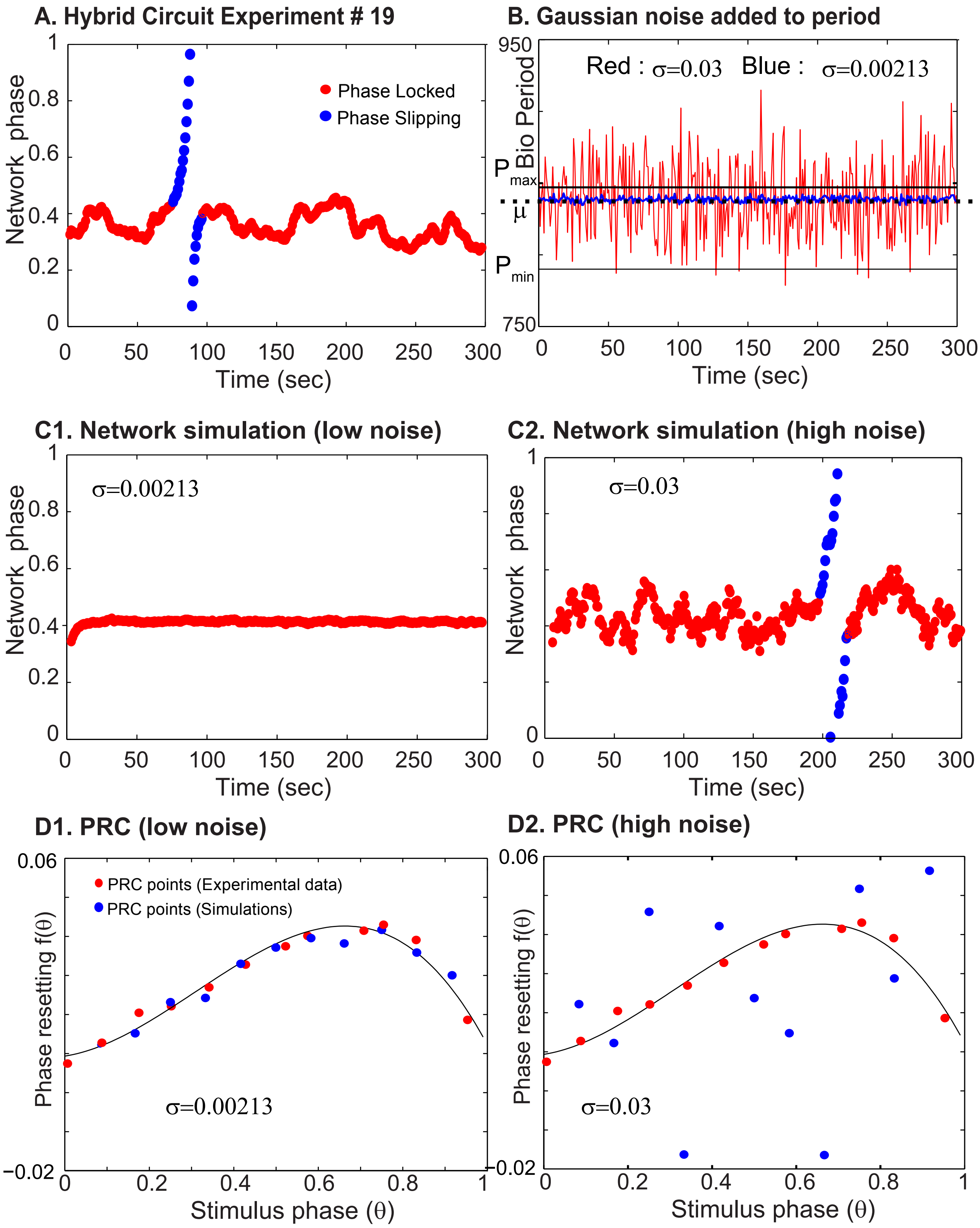

A. Hybrid Circuit Experiment # 19
Network phase
Phase Locked
Phase Slipping
Time (sec)
B. Gaussian noise added to period
Red : σ=0.03 Blue : σ=0.00213
Bio Period
P max
μ
P min
Time (sec)
C1. Network simulation (low noise)
σ=0.00213
Network phase
Time (sec)
C2. Network simulation (high noise)
σ=0.03
Network phase
Time (sec)
D1. PRC (low noise)
PRC points (Experimental data)
PRC points (Simulations)
Phase resetting f(θ)
σ=0.00213
Stimulus phase (θ)
D2. PRC (high noise)
Phase resetting f(θ)
σ=0.03
Stimulus phase (θ)
