# Supplementary material for: Slow Noise in the Period of a Biological Oscillator Underlies Gradual Trends and Abrupt Transitions in Phasic Relationships in Hybrid Neural Networks
Source: PLoS Comput Biol. 2014 May 15;10(5):e1003622. doi: 10.1371/journal.pcbi.1003622 (PMC4022488; doi:10.1371/journal.pcbi.1003622)
Supplement: Figure S2 — A parameter regime spanning a wide range of τ but a narrow range of σ fits both the PRC and hybrid circuit data for the OU model. The parameter grid was sampled at the red dots, and the parenthetical expressions indicate the squared error ratio for the simulated to experimental PRCs as well as the range of bifurcations that were observed in ten random simulations of the hybrid circuit. The blue ellipses indicate the parameter space where the ratio is near 1 and the bifurcation range includes zero, since these experiments were always phase locked. (A) Experiment 25, (B) Experiment 27, (C) Experiment 28, (D) Experiment 34. (PDF) [file pcbi.1003622.s002.pdf]

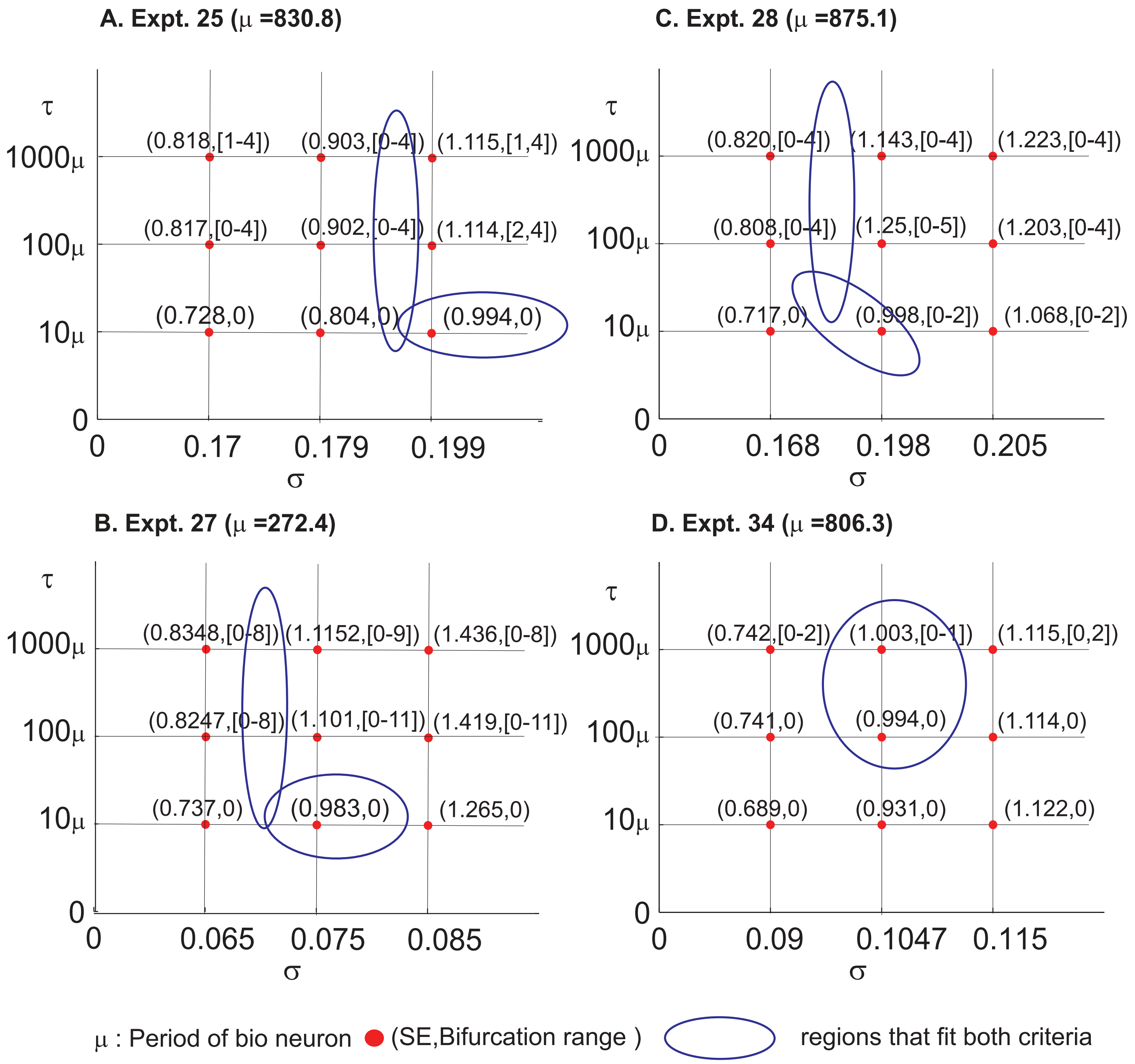
A. Expt. 25 (μ =830.8)
C. Expt. 28 (μ =875.1)
B. Expt. 27 (μ =272.4)
D. Expt. 34 (μ =806.3)
τ
σ
μ : Period of bio neuron
(SE,Bifurcation range)
regions that fit both criteria
