# Supplementary material for: Slow Noise in the Period of a Biological Oscillator Underlies Gradual Trends and Abrupt Transitions in Phasic Relationships in Hybrid Neural Networks
Source: PLoS Comput Biol. 2014 May 15;10(5):e1003622. doi: 10.1371/journal.pcbi.1003622 (PMC4022488; doi:10.1371/journal.pcbi.1003622)
Supplement: Figure S3 — Added Gaussian noise only picks up easily accessible, and sometimes questionable, bifurcations. A. Network phase of hybrid circuit for Experiment 14 with phase slipping (blue dots) punctuated by “sticky” phase locking (red dots). B. Simulations confirm brief episodes identified as phase locked. C. Interaction (ts-tr) curves just miss intersecting, so a small amount of change in the biological period can cause an intersection (and phase locking) to occur. D. Time course of the unobservable intrinsic period of the biological neuron during simulations of this experiment (blue trace). The top dashed line shows the initial (and mean period) whereas the solid horizontal lines indicate the values of the period between which an intersection exists in the ts-tr curves. (PDF) [file pcbi.1003622.s003.pdf]

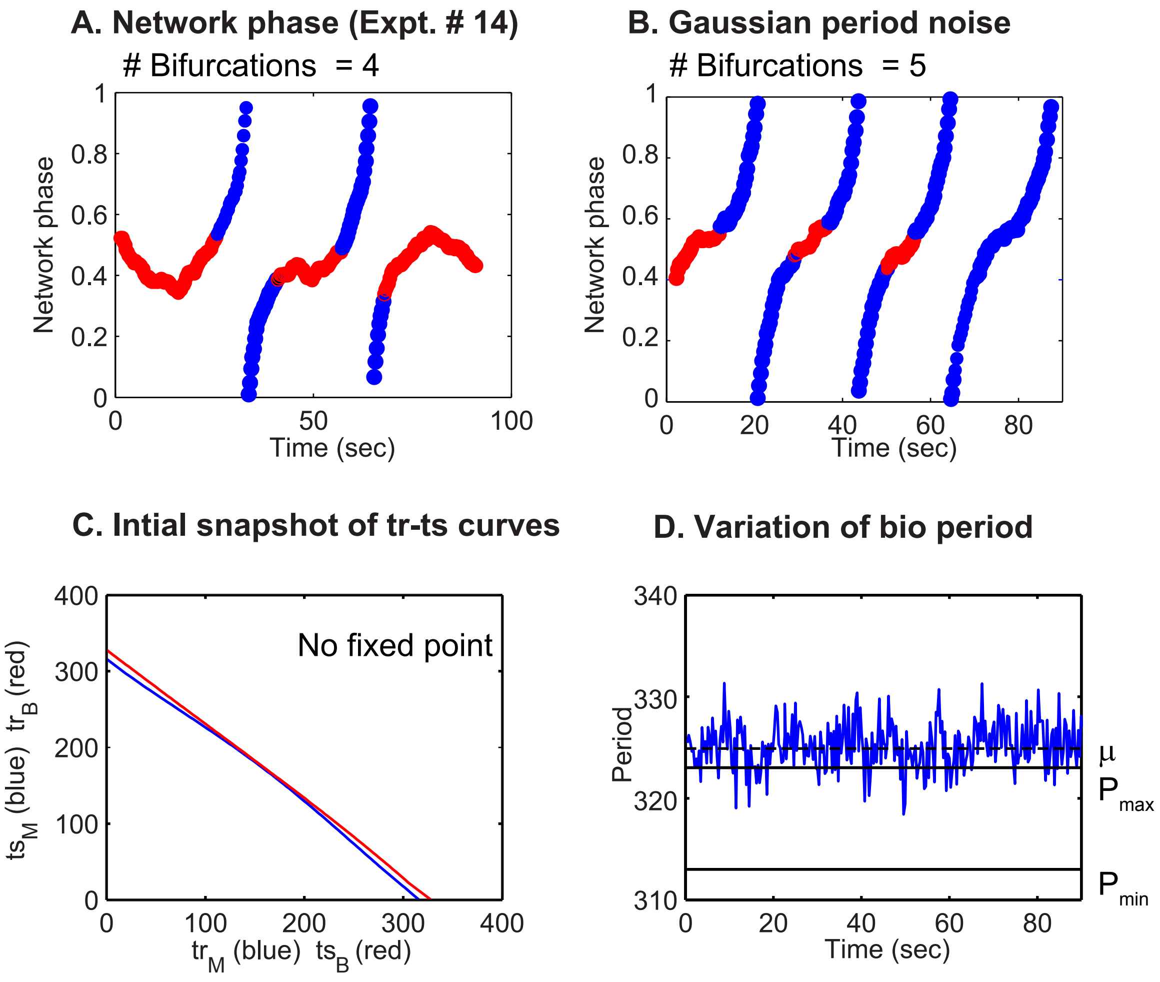
A. Network phase (Expt. # 14)
Bifurcations = 4
Network phase
Time (sec)
B. Gaussian period noise
Bifurcations = 5
Network phase
Time (sec)
C. Intial snapshot of tr-ts curves
No fixed point
ts_M (blue) tr_B (red)
tr_M (blue) ts_B (red)
D. Variation of bio period
Period
Time (sec)
μ
P_{max}
P_{min}
