# Supplementary figures and images for: Slow Noise in the Period of a Biological Oscillator Underlies Gradual Trends and Abrupt Transitions in Phasic Relationships in Hybrid Neural Networks
Source: PLoS Comput Biol. 2014 May 15;10(5):e1003622. doi: 10.1371/journal.pcbi.1003622 (PMC4022488; doi:10.1371/journal.pcbi.1003622)

**A. Hybrid Circuit Experiment 19**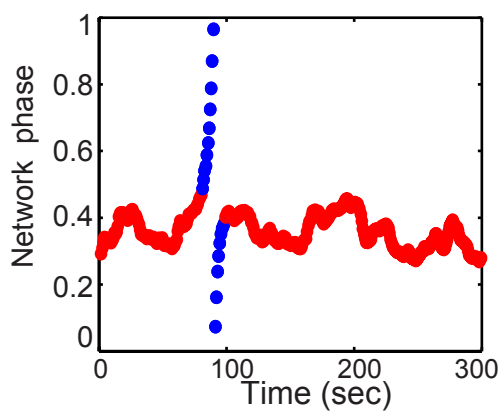**B. No mean reversion**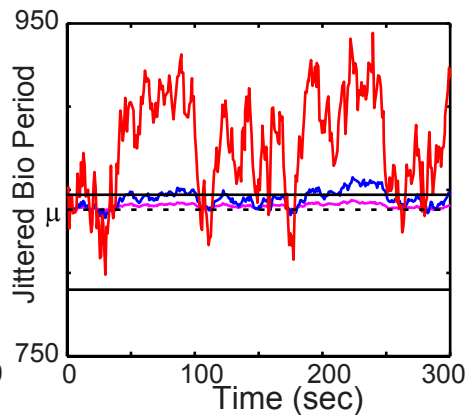**C. Autocorrelation**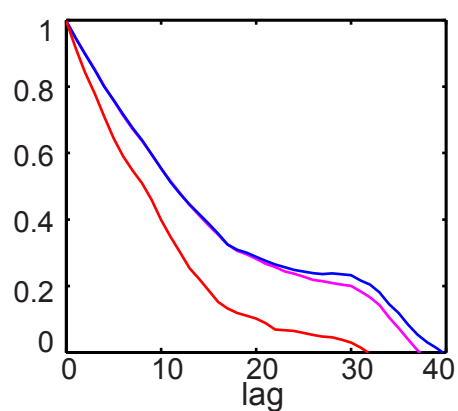**D1**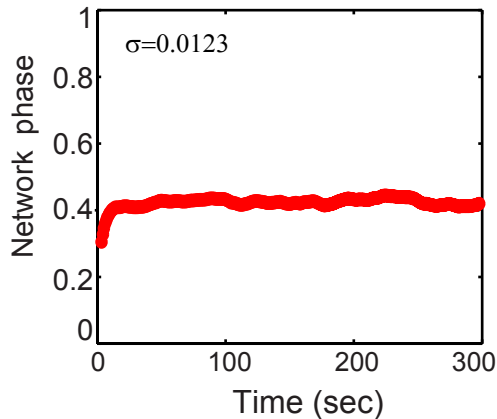**D2**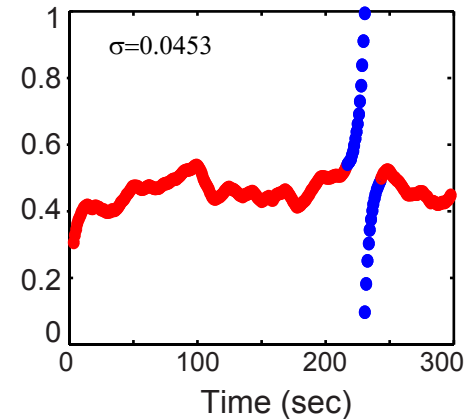**D3**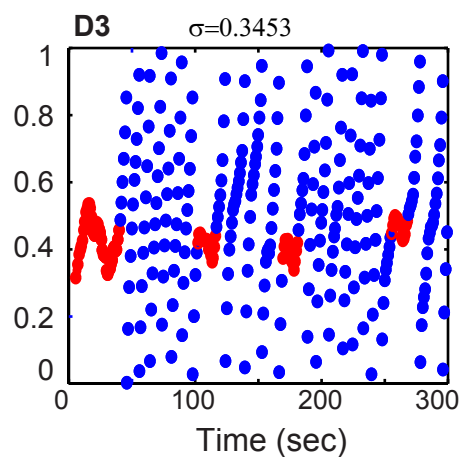**E1**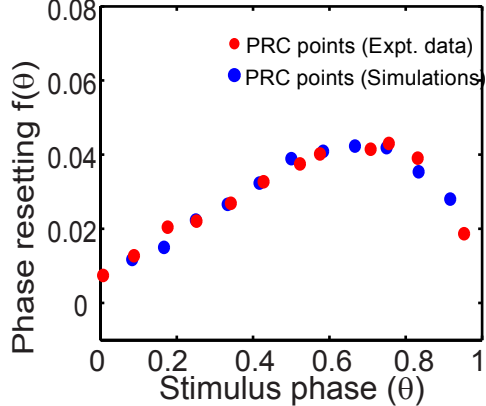**E2**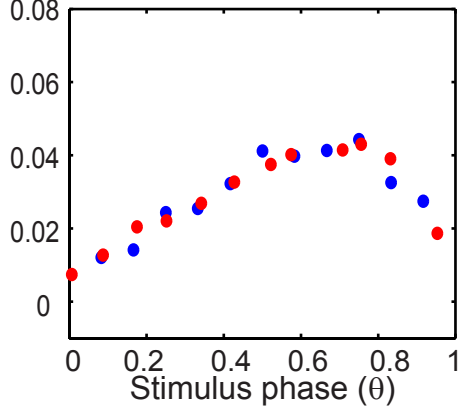**E3**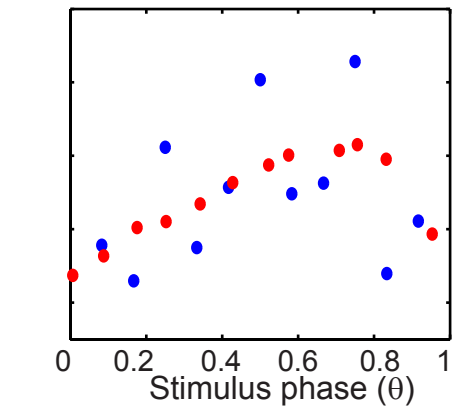

Supplement: Figure S4 — History dependence of the period without mean reversion is sufficient to mimic both the hybrid circuit data and the noise level in the PRC with the same parameter value. A. Network phase data replotted from Fig. 8A for experiment 19. B. Time course of the unobservable intrinsic period of the biological neuron during simulations of this experiment for σ = 0.0123 (magenta trace), σ = 0.0453 (blue trace) and σ = 0.3453 (red trace). The simulations used Eq. 2 without the term containing τ, so τ was effectively set to infinity.The center dashed line shows the initial period (but not the mean in this case), whereas the solid horizontal lines indicate the values of the period between which an intersection exists in the ts-tr curves (see Figure 7b). C. Autocorrelation values for the same three σ values as in B. D. Simulation of hybrid network for low noise (D1), medium noise (D2) and high noise (D3) case. E. Comparison of experimental (red dots) and representative simulated (blue dots) PRC measurements with low noise (E1) and medium noise (E2) and high noise (E3). (PDF) [file pcbi.1003622.s004.pdf]
