# Supplementary material for: Slow Noise in the Period of a Biological Oscillator Underlies Gradual Trends and Abrupt Transitions in Phasic Relationships in Hybrid Neural Networks
Source: PLoS Comput Biol. 2014 May 15;10(5):e1003622. doi: 10.1371/journal.pcbi.1003622 (PMC4022488; doi:10.1371/journal.pcbi.1003622)
Supplement: Text S1 — This supplementary material contains 1) a detailed explanation of the criteria for identifying phase slipping and phase-locked episodes, 2) a derivation of stability for the PRC-based map, and 3) a derivation of effective standard deviation of the period for noise added to the PRC. (PDF) [file pcbi.1003622.s005.pdf]

Supplementary Material

1) Data Analysis: Criteria for identifying phase slipping and phase-locked episodes

The first 10 cycles of the network phase (ϕ) after coupling was turned on were ignored to minimize the effect of transients on attractor dynamics. The network phase is determined in a stroboscopic manner in each cycle by taking a spike in the model neuron as reference point, then computing the network phase of the model neuron relative to the biological neuron as $\phi = ts_M/(ts_M+tr_M)$. The following automated algorithm for detecting episodes of phase-locking or phase slipping was applied to both the experimental data and the PRC-based simulations. Episodes that did not meet either criterion were classified as other. During a slipping episode, the magnitude of the relative phase difference increases constantly, indicating that one oscillator is faster than the other and always advancing in phase with respect to the other. Therefore a change in the slope of the relative phase difference terminated a slipping episode. In order to qualify as a slipping episode, the monotonic slope of network phase had to be maintained for an accumulated change in network phase equal to 0.4. This was calculated by adding all of the changes in network phase during a run of constant slope. During periods of sticking, or very slow rate of change in the network phase, noise could theoretically induce spurious changes in slope, so the algorithm allowed a small (magnitude of 0.01) change in phase in the opposite direction of the prevailing trend every 10 cycles in order to capture the global trend better. In order to determine phase locked episodes, episodes that were previously identified as slips were omitted. In the remaining data, if the minimum and maximum phase remained within 0.2 phase units of each other for 20 cycles, a phase locking episode was identified. The parameters in the algorithm described above were adjusted manually to ensure that the code could identify episodes in the experimental data that were obvious episodes of locking or slipping. In some cases it is not clear if the circuit is switching between phase locked and phase slipping modes versus simply slipping very slowly at a particular phase on each phase slipping episode.

2) *Derivation of stability for the PRC-based map*

Here we derive stability criteria given the tr-ts maps of a pair of coupled neurons. The inequality in terms of the slopes can be written as follows, with the slope of the (tr_M,ts_M) curve on the left and the slope of the (ts_B,tr_B) on the right:

$$\left|\frac{ts_M(\theta)-ts_M(\theta-\Delta\theta)}{tr_M(\theta)-tr_M(\theta-\Delta\theta)}\right| > \left|\frac{tr_B(\theta)-tr_B(\theta+\Delta\theta)}{ts_B(\theta)-ts_B(\theta+\Delta\theta)}\right|$$

The difference in the sign of Δθ results because θ increases to the left on the (tr_M,ts_M) curve and to the right on the (ts_B,tr_B) curve. Using a taylor series approximation to first order and simplifying we obtain

$$\left|\frac{1}{1-f_M'(\theta)}\right| > \left|1-f_B'(\theta)\right|$$

Examining all cases for the signs of the terms on each side, we obtain

$1 > (1-f_B'(\theta))(1-f_M'(\theta)) > -1$ which is identical to the stability constraint for two pulse coupled oscillators given in Dror et al 1999.

3) *Derivation of effective standard deviation of the period*

Noise added to PRC

The perturbed period P_1 in terms of the unperturbed period P_0 is $P_1 = P_0 (1 + f(\theta) +\sigma X)$. However, in the presumed absence of noise one would infer an observable intrinsic period $P_{0,i}$ as follows:

$P_1 = P_{0,i} (1 + f(\theta)) = P_0 (1 + f(\theta) + \sigma X)$

Rearranging

$P_{0,i} (1 + f(\theta)) = P_0 \cdot (1 + f(\theta)) + P_0 \sigma X$

$(P_{0,i} - P_0) (1 + f(\theta)) = P_0 \sigma X$

$P_{0,i} - P_0 = X\sigma P_0 /(1 + f(\theta))$

Note that *f(θ)* is not constant, but depend on the phase at which an input is received. Therefore the distribution of the inferred change in the intrinsic period is no longer strictly Gaussian, but the maximum effective standard deviation of intrinsic period change can be considered to be approximately bounded as follows:

$$\sigma_{eff} = \frac{P_0\sigma}{1+f_{(\min)}(\theta)} \text{ since } \boldsymbol{\sigma_{eff}} = \frac{\boldsymbol{\sigma}}{\mathbf{1}+\boldsymbol{f}_{(\mathbf{min})}(\boldsymbol{\theta})} > \frac{\boldsymbol{\sigma}}{\mathbf{1}+\boldsymbol{f}_{(\mathbf{max})}(\boldsymbol{\theta})},$$

where $f_{min}(\theta)$ and $f_{max}(\theta)$ are minimum and maximum of the PRC.

References:

Dror RO, Canavier CC, Butera RJ, Clark JW and Byrne JH (1999). A mathematical criterion based on phase response curves for the stability of a ring network of oscillators. *Biol. Cybernetics,* 80:11-23.
